# Disease-dependent interaction policies to support health and economic outcomes during the COVID-19 epidemic

**DOI:** 10.1016/j.isci.2021.102710

**Published:** 2021-06-10

**Authors:** Guanlin Li, Shashwat Shivam, Michael E. Hochberg, Yorai Wardi, Joshua S. Weitz

**Affiliations:** 1 Interdisciplinary Graduate Program in Quantitative Biosciences, Georgia Institute of Technology, Atlanta, GA, USA; 2 School of Physics, Georgia Institute of Technology, Atlanta, GA, USA; 3 School of Electrical and Computer Engineering, Georgia Institute of Technology, Atlanta, GA, USA; 4 ISEM, Université de Montpellier, CNRS, IRD, EPHE, Montpellier, France; 5 Santa Fe Institute, Santa Fe, NM, USA; 6 School of Biological Sciences, Georgia Institute of Technology, Atlanta, GA, USA

**Keywords:** Virology

## Abstract

Lockdowns and stay-at-home orders have partially mitigated the spread of Covid-19. However, en *masse* mitigation has come with substantial socioeconomic costs. In this paper, we demonstrate how individualized policies based on disease status can reduce transmission risk while minimizing impacts on economic outcomes. We design feedback control policies informed by optimal control solutions to modulate interaction rates of individuals based on the epidemic state. We identify personalized interaction rates such that recovered/immune individuals elevate their interactions and susceptible individuals remain at home before returning to pre-lockdown levels. As we show, feedback control policies can yield similar population-wide infection rates to total shutdown but with significantly lower economic costs and with greater robustness to uncertainty compared to optimal control policies. Our analysis shows that test-driven improvements in isolation efficiency of infectious individuals can inform disease-dependent interaction policies that mitigate transmission while enhancing the return of individuals to pre-pandemic economic activity.

## Highlights

• We develop a feedback approach to mitigate COVID-19 and enable safer return-to-work
• Feedback policies show greater robustness to uncertainty compared to optimal control
• Return-to-work is accelerated by large-scale viral and serological testing
• Large-scale testing guides targeted changes in behavior rather than en-masse lockdowns

## Graphical abstract

**Figure:**
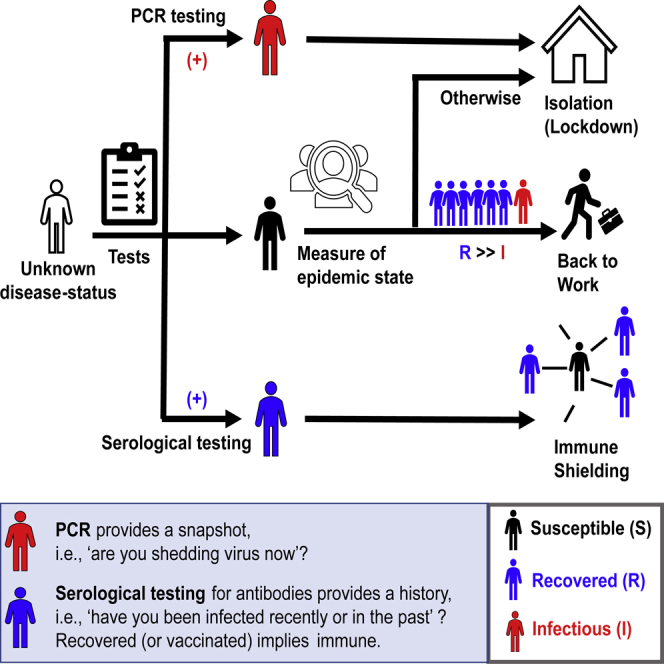

## Introduction

As of 7 March 2021, more than 116,166,652 cases of coronavirus disease 2019 (COVID-19) have been reported worldwide with more than 2,582,528 deaths globally (World Health Organization, 2021). Starting at the reported origin of the pandemic in Wuhan, China, control measures have been implemented in most countries where outbreaks have occurred (Flaxman et al., 2020; Chinazzi et al., 2020; Wells et al., 2020; Cowling et al., 2020; Ferguson et al., 2020; Kraemer et al., 2020). Multiple public health strategies are being deployed to slow outbreaks, and although recommendations always include social distancing and isolation of confirmed cases, the full spectrum of measures and levels of adherence differ from country to country, making assessments of strategy efficacy difficult (see (Flaxman et al., 2020), controversy surrounding (Zhang et al., 2020)).

The non-pharmaceutical control strategies for COVID-19 largely follow those employed in previous viral epidemics, including SARS, Ebola and MERS. Initial strategies can be broadly grouped into mitigation and suppression, where the former attempts to preserve essential health care services and contain morbidity and mortality, whereas the latter imposes more severe, emergency restrictions to prevent health care system collapse and provide conditions for easing-off toward less intense mitigation strategies (Walker et al., 2020). Both mitigation and suppression approaches carry considerable social and economic costs, meaning that policymakers and the public at large only adopt them for short time periods (OECDa, 2020). A problem is that control measures have often been applied irrespective of an individual's disease status (and/or likely infection risk severity) and are driven, in part, by the absence of information-driven alternatives.

Hence, distinct from lockdowns, there is an increasing interest in implementing population-wide prevention methods that decrease transmission risk while enabling economic re-engagement. Examples of such measures include mask-wearing (Greenhalgh et al., 2020; Chu et al., 2020), contact-free interactions (Brynjolfsson et al., 2020), and restructuring of physical spaces (Bodkin et al., 2020). The use of masks, in particular, has been shown to be effective at reducing respiratory transmission of SARS-CoV-2, particularly when individuals in a potentially infectious interaction routinely wear them (Feng et al., 2020; Cheng et al., 2020). These population-wide measures still carry uncertainty since individuals are expected to behave uniformly irrespective of their disease status. COVID-19 screening provides a complementary route, and despite costs may confer both health and economic benefit (Atkeson et al., 2020) to overcome the negative impact on the economy due to pandemic-related shutdowns (Baqaee et al., 2020). As the scale of COVID-19 testing has increased, jurisdictions may also have an opportunity to consider implementing tactical mitigation strategies informed by testing.

Testing for infected status can, in theory, be used to initiate targeted isolation, identification and tracing of contacts, quarantining of contacts, and then selected testing of contacts (Ferretti et al., 2020). If done rapidly and at scale, this kind of targeted PCR-based testing can provide early detection of cases and help break new chains of transmission (He et al., 2020; Yong et al., 2020; Gibson et al., 2021; Denny, 2020; Specht et al., 2021). The effectiveness of contact-tracing based control strategies hinges on the accurate identification and isolation of exposed and infectious cases. Intensive and stringent testing and isolation policies have even enabled some countries to reopen (Coglianese and Mahboubi, 2021). Slow return of test results (primarily) and false negatives (as a secondary factor) limit the effectiveness of test-based control policy (Larremore et al., 2021). A complementary tactic is the strategic deployment of immune individuals to effectively dilute transmission events (Kraay et al., 2020; Weitz et al., 2020a). Similar to the identification of infected individuals through PCR tests, immunity to SARS-CoV-2 is assessed through serological testing for protective antibodies (Amanat et al., 2020; Krammer and Simon, 2020) or presumed through vaccination. The overall benefits of shielding, as quantified via detailed modeling studies, have been shown to outweigh the potential costs associated with false positives insofar as high-quality tests are utilized (Kraay et al., 2020). Population-wide interventions, testing efforts, the selective confinement or deployment of people contingent on their infection status, and inaccuracies and limited adherence to policies, combine to create a challenging landscape for the persistent control of outbreaks (Fauci et al., 2020).

Non-pharmaceutical COVID-19 control until effective vaccines become widely available will necessarily involve periods of reduced social and economic activity; i.e., ‘business, but not as usual’. Control efforts are already generating hardship and could in the longer-term result in social unrest and increased mortality (OECD, 2020b; Douglas et al., 2020; Hsiang et al., 2020). Here we confront a joint problem: how to identify policies that aim to reduce fatalities arising from COVID-19 while also enabling economic engagement. First, we use optimal control to assess both health and economic outcomes in an susceptible exposed infectious recovered (SEIR) disease model framework. There is a substantial and growing literature on optimal control for COVID-19, the bulk of which focuses on non-personalized release policies or policies that target age- or risk-stratified groups (Bonnans and Gianatti, 2020; Flaxman et al., 2020; Ferguson et al., 2020; Walker et al., 2020; Morris et al., 2021; Zhao and Feng, 2020). Here, we identify optimal control policies to modulate interaction rates based on disease – unifying prior efforts centered on isolation and shield immunity. We find that intermediate policy outcomes can do nearly, as well as strict public health scenarios, without incurring the severe costs as suppression-centered policies. However, optimal controls can be fragile, when applied in practice given that they rely on time-rather than state-based interventions; the consequence of mistiming interventions can be severe (Morris et al., 2021), Hence, guided by the optimal control analysis, we identify state-dependent policies similar to feedback control that provide actionable guidance for individual behavior. As we show, using population-wide PCR testing for infection alongside immune status can reduce COVID-19 transmission while enabling more individuals to return-to-work sooner and with fewer restrictions than would otherwise be possible.

## Results

### Optimal control framework for state-dependent contact rates policies that balance public health and socioeconomic costs

**Figure 1:**
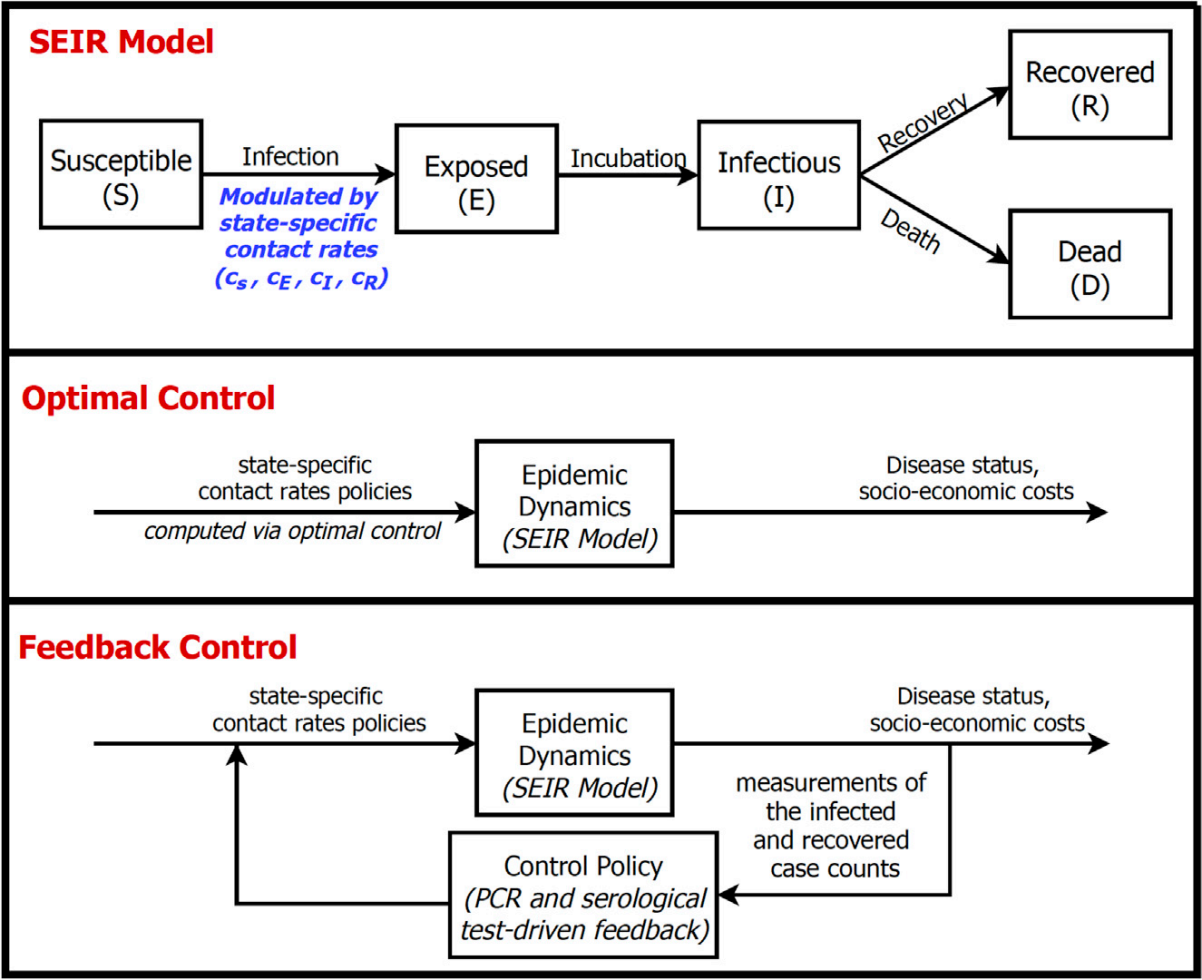
Epidemic dynamics with optimal and feedback control of disease status-driven contact rates (*Top) SEIR model schematic in which the force of infection is modulated by state-specific contact rates, see text and*supplemental information*for details. (Middle) Diagram of optimal control approach: contact rates are pre-specified given model structure and estimate of parameters and current conditions. (Bottom) Diagram of feedback control approach: contact rates are updated in real-time based on measurements of the infected and recovered/immune case counts via testing surveillance.*

We develop an optimal control framework to identify policies that address the tension between decreasing contacts (that reduce new infections) with increasing contacts (that are linked to socioeconomic benefits). We represent the epidemic using a SEIR nonlinear dynamic model (see supplemental information for complete details; see Figure 1). In doing so, the force of infection is influenced by state-specific contact rates *c*_*S*_*, c*_*E*_*, c*_*I*_ and *c*_*R*_ for susceptible, exposed, infectious and recovered/immune individuals, respectively – these different levels form the basis for a control policy that directs individuals to interact at different levels depending on their test status.

In the optimal control framework, a set of state-specific contact rates are identified that minimize the appropriately weighted sum of what we term ‘public health’ and ‘socioeconomic’ costs. Public health costs are quantified both by average infected levels and cumulative deaths. Socioeconomic costs are quantified in terms of reductions in the total rate of interactions and by shifts in state-specific contact rates. The optimal control ‘solution’ is then a time-dependent set of disease-specific rates which are both shaped by and shape the epidemic itself (see supplemental information for details on the gradient projection algorithm used to identify the solution). Note that we constrain the contact rate of exposed individuals to be equal to that of susceptible individuals given the challenges of timely identification of exposed individuals who are not yet infectious (and presumably have insufficient viral titer to be identified using screening tests; an issue we return to in the discussion). Finally, we utilize the parameter ξ to regulate the relative importance of costs associated with death and spread of infection vs. socioeconomic impact.

**Figure 2:**
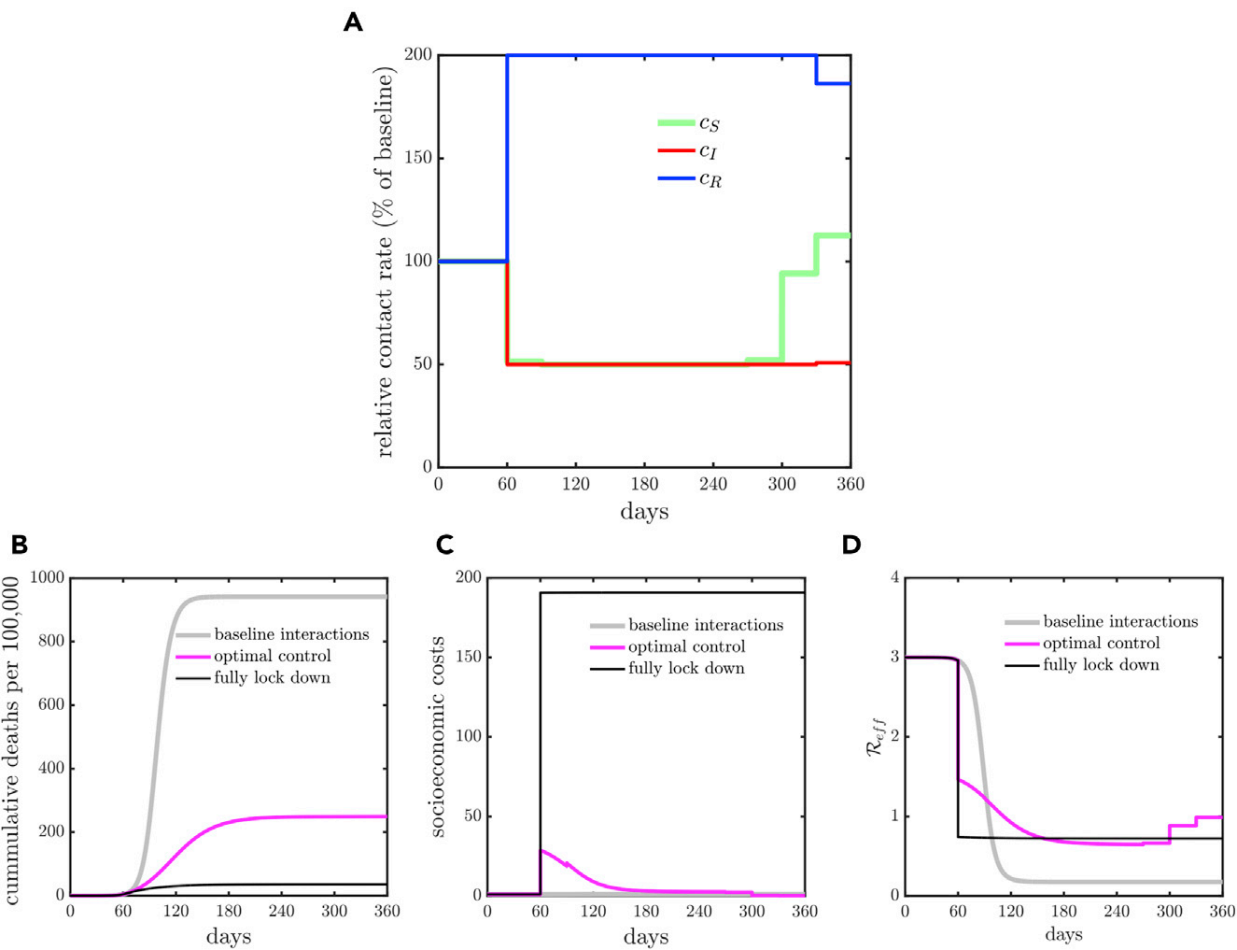
Health and economic outcomes associated with COVID-19 dynamics Comparison of health and economic outcomes of COVID-19 given various interventions: baseline interactions (i.e., no intervention); optimal contact rate intervention (balance both health and economic outcomes) and fully lock down intervention (applied to all the subpopulations) with 75% isolation efficiency. (A) The optimal contact rate relative to the baseline contact rate (denoted as 100%) with 50% isolation effectiveness and shield immunity level 2 (B) Cumulative deaths (health outcome) during the epidemic. (C) Socioeconomic costs (economic outcome) during the epidemic. (D) Measure of effective reproduction number ($R_{\text{eff}}$) for different interventions during the epidemic.

Figure 2 shows the results of comparing a baseline outbreak (i.e., neglecting public health costs, given weighting parameter ξ = 0) to a full lockdown scenario (i.e., neglecting socioeconomic costs with 75% isolation for all, ξ >> 1) and a balanced scenario with optimized contact rates (i.e., corresponding to ξ = 1). At the time intervention starts (without any intervention in first 60 days), the outbreak has an ongoing incidence of 19 (per 100,000) per day, prevalence of 0.4%, and a cumulative infection level of 0.8%. As shown in Figure 2, in the baseline scenario, the disease spreads through the population leading to 94% cumulative infection (as expected given strength-size relationships for R_*0*_ *=* 3). In contrast, a full lockdown scenario with 75% reduction in contact rates of all individuals after 60 days leads to a total outbreak size of 4% of the population. The optimal control solution in the balanced case ξ = 1 reveals a potential route to jointly address public health and socioeconomic cost. From the perspective of public health, the optimal control solution leads to 25% cumulative infections. In addition, the socioeconomic costs in the optimal control case are higher in the short-term but approach that of the baseline scenario in the long-term. Indeed, the effective reproduction number identified via an optimal control framework in the balanced scenario gradually reduces to sub-critical levels (close to an effective reproduction number, $R_{\mathrm{eff}}$ = 0.75) while gradually relaxing controls over time. The optimal control solutions are shown in Figure 2A. The optimal control solutions differ based on disease status, recovered/immune individuals elevate their interactions, infectious individuals isolate, and susceptible individuals lockdown before gradually returning to pre-lockdown levels.

### Personalized, test-based optimal control policies and their impact on public health and socioeconomic outcomes

**Figure 3:**
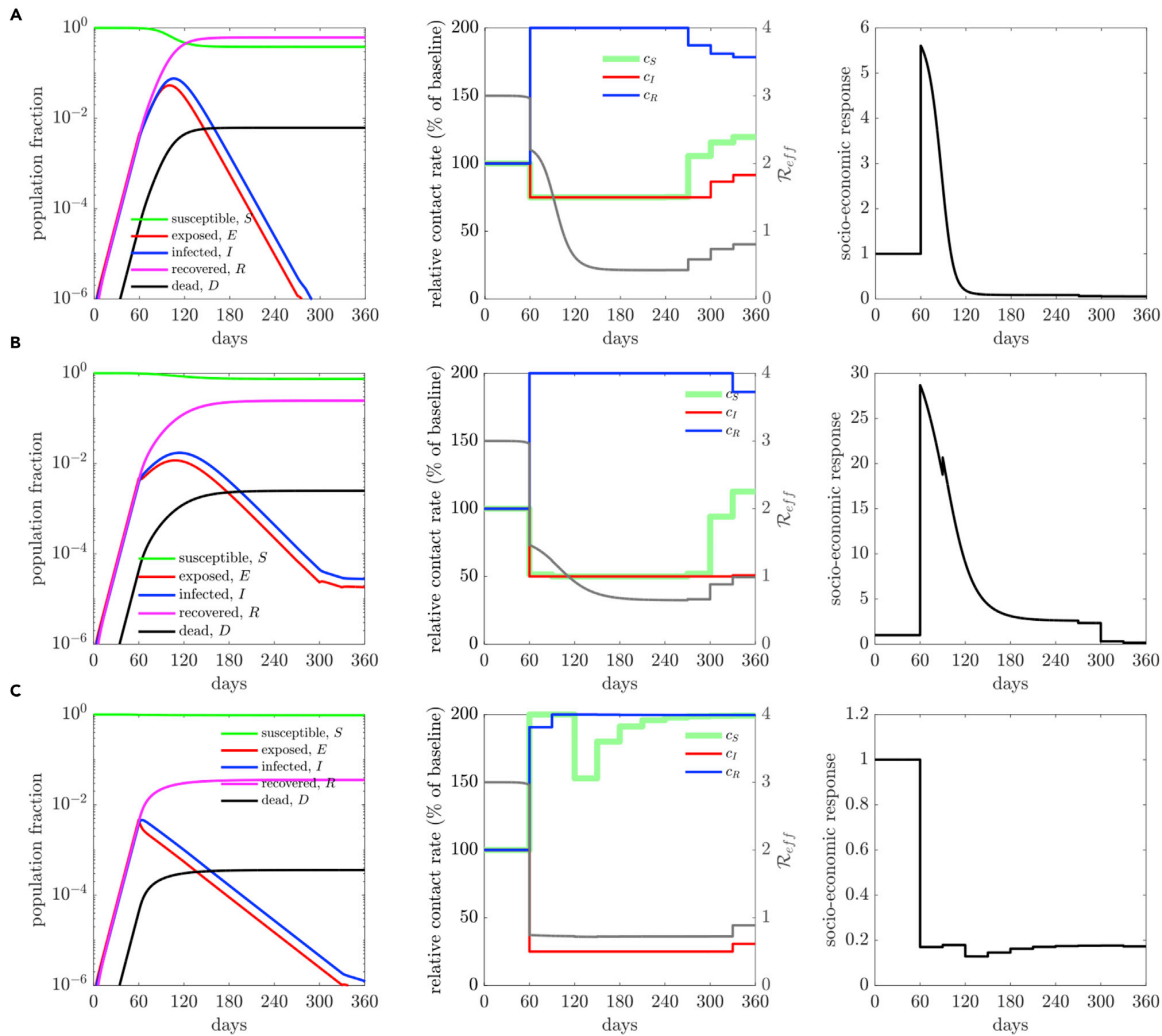
Epidemic dynamics and socioeconomic response with optimal contact rate interventions SEIR dynamics with contact rate interventions for various isolation efficiencies, (A) 25% isolation efficiency; (B) 50% isolation efficiency and (C) 75% isolation efficiency. The relative importance (ξ) is 1 for all the cases (A), (B), and (C). The contact rate interventions start at 60 days, and people follow baseline (or normal) interactions before that. For all the isolation efficiency scenarios (three rows), the left panel shows the population dynamics given the optimal contact rate (related to the baseline contact rate, with 100% as baseline) shown in the middle panel. The gray curve in the middle panel represents the measure of corresponding effective reproduction number ($R_{\text{eff}}$). The right panel shows the corresponding socioeconomic costs. See supplemental information for additional scenarios.

In order to explore the mechanisms identified by the optimal control framework, we systematically modulate the effectiveness of isolation and evaluate its effect on the state-dependent optimal contact rates and disease dynamics. In practice, isolation effectiveness is influenced by availability, accuracy, and speed of testing, as well as fundamental limitations on an individual's ability to isolate (which can vary with socioeconomic and other factors). Figures 3A–3C evaluate low, medium, and high efficiency of isolation spanning 25%, 50%, and 75% reduction in the contact rates of infected individuals, respectively. As is evident, the optimal control solutions for state-dependent contact rates vary significantly with isolation effectiveness; suggesting that COVID-19 response policies that can vary with disease status may open up new possibilities to balance public health and socioeconomic outcomes.

First, in the low (25%) or medium (50%) effectiveness cases, susceptible, exposed, and infectious individuals adopt the maximal level of isolation. Inefficient isolation of infectious individuals elevates risks of new transmission that are not outweighed by socioeconomic benefits. Notably, the optimal control solution includes an *elevated* level of interaction by recovered/immune individuals. This finding recapitulates ‘shield immunity’ (Kraay et al., 2020; Weitz et al., 2020a), insofar as recovered/immune individuals are protected from re-infection over the course of the intervention. The elevated contacts of recovered/immune individuals have multiple effects: both diluting interactions by susceptibles (and reducing transmission risk) and by increasing socioeconomic activity. In contrast, for sufficiently high levels of isolation efficiency (75%), the optimal control solutions suggest there is no need for a general lockdown. Instead, the combination of infected case isolation and shielding by the subpopulation of recovered/immune individuals is sufficient to rapidly reduce and contain $R_{\mathrm{eff}}$ below 1, leading to a decreasing number of new infections. We note that irrespective of isolation effectiveness, balancing public health and economic outcomes drives $R_{\mathrm{eff}}$ below 1, but not necessarily to 0 (albeit, given the constraints imposed by lockdown efficiency, such an extreme reduction may not even be possible), and eventually increased immunity permits an easing-off in restrictions yielding an increase in $R_{\mathrm{eff}}$ (Hochberg, 2020).

### Sensitivity of optimal control approach to mistimed implementation of policies

Despite its potential to balance public health and socioeconomic costs, a central drawback of optimal control solutions is the potential exponential growth of errors. Given the fact the COVID-19 dynamics are only partially observed (with significant uncertainty in the actual state), application of a policy requires an estimate of the time since epidemic initiation, what we term ‘epidemic age’. In order to evaluate the sensitivity of optimal control policies due to mistiming, we first computed the optimal control policy for a system one month after an outbreak. However, instead of implementing the policy matched to the actual epidemic age, we enforce the optimal control policy 30 days later, i.e. at the end of 60 days after the start of the outbreak. Figure S7 shows the difference in the mistimed control policy vs. the optimal control policy; as is evident the mistimed policy relaxes stringent lockdown when the optimal policy continues to lockdown. As a consequence the total deaths are far higher for 25% and 50% isolation efficiency (see Table 1). The mistimed policy, in effect, biases the system toward minimizing socioeconomic rather than public health costs. This significant difference in performance metrics demonstrates the potential shortcomings of implementing a policy based on optimal control. However, we note that with a stringent isolation efficiency, delays are less problematic. The reason is that with efficient infected case isolation, both the mistimed and optimal control policy could enable nearly all individuals to work, given that $R_{\mathrm{eff}}$ is held below 1 by infection isolation on its own.

**Table 1:** Comparison between optimal control approach for contact policy with and without delay

Optimal control approach	Without delay		With delay	
Efficiency of isolation (shielding = 2)	Total deaths (per 100,000 individuals)	Working fraction	Total deaths (per 100,000 individuals)	Working fraction
25%	600	90.95%	720	94.96%
50%	250	67.13%	510	83.65%
75%	40	99.92%	40	99.95%

The comparisons are made for isolation efficiencies of 25%, 50% and 75%, with performance metrics of total deaths (per 100,000 individuals) and working fraction. The total death is significantly higher for the system with delay when the isolation efficiency is not 75%, suggesting poor robustness to delay. Feedback control policy for balancing public health and socioeconomic costs.

Despite its fragility, we identify common features of the optimal control policy given variation in the effectiveness of infectious case isolation. First, the optimal control policy minimizes infected contact rates. The optimal control solutions also robustly identify an immune shielding strategy such that recovered/immune individuals elevate their interactions to the maximum possible relative to baseline. Importantly, differences in the optimal control policy are primarily centered on identifying a switch point in contact rate level for the susceptible population. From Figures S4–S6, we observe the switch point as a function of time, showing that irrespective of isolated case effectiveness and shield immunity constraints, the increase in susceptible contact rates happens later in the lockdown period. Switchover points correspond to times when the infection prevalence is relatively low compared to the recovered/immune population. This observation provides the basis for a feedback, rather than optimal, control policy.

We propose the use of a feedback control policy adapted from emergent features of the optimal control policy solutions: (i) infectious individuals isolate as far as is possible; (ii) recovered/immune individuals increase their activities as much as possible, i.e., akin to shield immunity. Hence, we set out to identify a system-dependent change in the contact rate of susceptible individuals, separating lockdowns vs. return-to-work. In practice, we identify a critical curve in *I-R* plane (i.e., infected-recovered cases plane) via a genetic algorithm, such that the recommended behavior of susceptible individuals is dictated by surveillance-based estimates of infectious and recovered/immune individuals (see supplemental information for more details).

**Figure 4:**
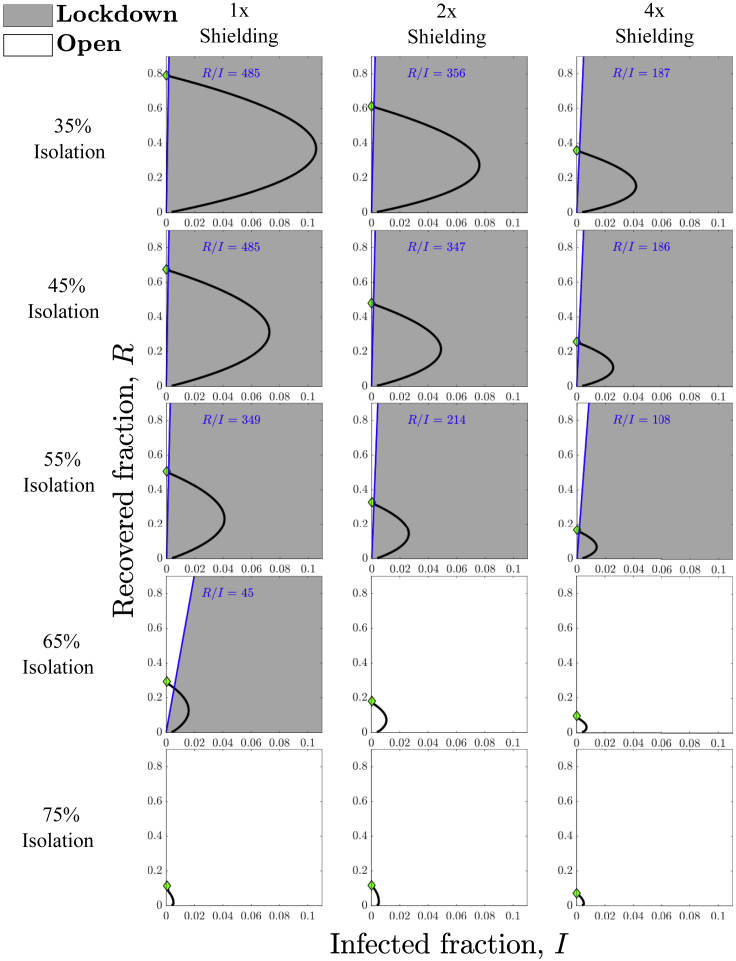
Heuristic state feedback intervention policies varying with isolation efficiency (rows) and shielding levels (columns) In each panel, the trajectory is noted in black with the final state as green diamond. An optimal line divides the plane into two regions which determines the optimal contact rate for the susceptible population for the current infected and recovered cases. The optimal policy in the dark gray region is lockdown and open in the white region. The phase plots show the dynamics of the infected and recovered case fractions over the period of 360 days, while applying the control strategy described above in the absence of shielding and for shielding levels of 2 and 4 respectively. For the case of isolation efficiency of 75%, no lockdown is needed at all for the susceptible population. See supplemental information for a larger set of plots with increments of 5% in isolation efficiency and for shielding levels of 2, 3, 4, and 5.

Figure 4 summarizes the results of the feedback control policy. From a policy perspective, the feedback control policy identifies a switch between lockdown and return-to-work when there are significantly more recovered/immune individuals than infectious individuals. The timing of the return from lockdowns is accelerated given increases in isolation efficiency (rows) with additional benefits from the implementation of shield immunity (columns). Typically, the transition between lockdown and re-openings occur when circulating case levels are low relative to recovered/immune individuals. The critical ratio of recovered to infectious individuals decreases as isolation increases. Notably, when there are sufficiently high levels of isolation of infectious individuals then the optimal feedback policy suggests that no lockdown is required (note the entirely ‘white’ regions in the bottom row). In the discussion section, we provide additional context on the potential impacts of vaccination on this central finding.

Critically, the performance of the test-driven feedback policy is nearly identical for the performance metrics with or without mistiming (see Table 2). This finding implies that state-based approaches will be less likely to have exponentially mistimed applications, and reinforces the need for population-scale testing for both active infections and recovered/immune individuals. To examine the robustness of the feedback strategy in terms of model mis-specification, we consider three cases in which the isolation efficiency is unbiased, overestimated (by ∼10%) and underestimated (by ∼10%) relative to the true value. We then compare the total deaths and working fraction of a feedback strategy based on these (potentially incorrect) estimates of the isolation efficiency. We find that the feedback strategy is robust to such mis-specification, particularly when isolation efficiency is high or low (see Figure S11 for details). We also note that a simple policy with only two states – “lockdown” and “open”, respectively, corresponding to minimum and baseline contact rates for the susceptible cases, would be easier to implement than one with continuous “phases” or state changes. In Figures S9 and S10, we document the generalizability of results given variation in infected case isolation and the level of shield immunity.

**Table 2:** Comparison between feedback control approach for contact policy with and without delay

Feedback control approach	Without delay		With delay	
Efficiency of isolation (shielding = 2)	Total deaths (per 100,000 individuals)	Working fraction	Total deaths (per 100,000 individuals)	Working fraction
25%	620	89.98%	620	89.94%
50%	250	62.40%	250	62.12%
75%	30	99.90%	30	99.90%

The comparisons are made for isolation efficiencies of 25%, 50% and 75%, with performance metrics of total deaths and working fraction. Both performance metrics are nearly identical for all efficiencies, suggesting no significant effect of delay on the system.

## Discussion

We have developed a linked series of optimal and feedback control analyses to evaluate the effectiveness (and benefits) of modifying contact rates for managing the COVID-19 pandemic from both health and economic perspectives. Throughout, our central goal was to optimize the interactions between individuals based on disease status so as to achieve a defined balance between public health and economic outcomes. By explicitly incorporating contact rates as the control variables in an SEIR model, we were able to identify optimal control policies that could, in theory, significantly reduce expected infections (and fatalities) while reducing the negative socioeconomic costs of sustained lockdowns. Optimal control policies are unlikely to be applied in practice, given the potential exponential mis-specification of policies over time. Hence, we leveraged insights from the optimal control solutions to guide a feedback control approach that performs nearly, as well as the optimal control approach with significant improvements in robustness given uncertainty in estimating the epidemic state. Collectively, our control policies indicate that infected individuals should be isolated (as effectively as possible), recovered/immune individuals should be encouraged to return-to-work (given benefits accrued via shield immunity), while the release of other individuals from lockdown should be guided by the epidemic state. The transition from lockdown to return-to-work occurs when circulating case-loads are far lower than recovered case counts; with the scope of the epidemic sharply controlled by infection case isolation. A combination of policies, e.g., mask-wearing, physical distancing, will help to reduce transmission risk for individuals who do return-to-work.

A salient point that emerged from the control analysis is the benefit of reducing interactions by infectious individuals and increasing interactions by recovered/immune individuals. In doing so, it is critical to note that the conception of the model *preceded* the availability of vaccines. The increase of activity by recovered/immune individuals effectively dilutes risky contacts between susceptible and infectious individuals. We contend that this principle of shield immunity is also relevant when individuals are vaccinated, and therefore move from susceptible directly to the recovered/immune category. Increases in vaccination may provide opportunities to reduce risk for susceptible individuals, beyond benefits accrued by susceptible depletion alone. We recognize that adopting policies that include individual disease status are likely to raise both privacy and ethical concerns (Phelan, 2020; Norheim, 2020). Yet, given the slow rate of vaccine dissemination, we suggest that the absence of action-taking that could increase protection to those yet to be vaccinated also comes at a public health and socioeconomic cost. Even now, more than a year after the identification of the first SARS-CoV-2 case, we remain closer to the beginning than the end of the Covid-19 pandemic. As we have shown, accelerating the slowdown of transmission while restoring economic activity may be enabled by both personalized, test-driven, policies as the basis for mitigation that reduce risk for all.

### Limitations of the study

The SEIR framework used as the basis for the present control study is intentionally simplified. The epidemic model does not account for process or observational noise, analytic test features, heterogeneity, stratified risk, asymptomatic cases, and detailed elaboration of severe cases. By reducing the model complexity, we have tried to shed light on the general problem of balancing public health with socioeconomic outcomes. In doing so, we have highlighted a middle ground between dichotomous outcomes that focus on public health or socioeconomic costs solely. We recognize that extensions and applications of the present work will require consideration of additional epidemic complexities (e.g., asymptomatic transmission) and additional evaluation of joint public health and economic costs (e.g., arising from hospitalization burden). Translating the present findings into practical use will also require improved assessments of the ways in which behavior is influenced by awareness and communication of the pandemic state (Weitz et al., 2020b*;* Franco, 2020), in addition to evaluating the influences of formal implementation of policy campaigns.

## STAR★methods

### Key resources table

**Table:** 

REAGENT or RESOURCE	SOURCE	IDENTIFIER
**Software and algorithms**		

Genetic Algorithm in Global Optimization Toolbox, MATLAB R2019a	MathWorks, Natick MA	https://www.mathworks.com/products/global-optimization.html
A MATLAB implementation of our dynamic model	This paper	https://github.com/WeitzGroup/COVID_Control_2020

### Resource availability

#### Lead contact

Further information and requests should be directed to and will be fulfilled by the lead contact Joshua S. Weitz (jsweitz@gatech.edu).

#### Materials availability

This study did not generate new unique reagents.

#### Data and code availability

All simulation and figure codes used in the development of this manuscript are available at https://github.com/WeitzGroup/COVID_Control_2020.

### Method details

Please find method details in the file “Supplemental procedures”.

## Supporting citations

The following reference appears in the Supplemental information: Anderson and May, 1992; Kirk, 2004; Liberzon, 2011; Ngonghala et al., 2020; Polak, 2012; The MathWorks, 2020; Whitley, 1994.

## Supplemental information

Document S1. Supplemental procedures, Figures S1–S11, and Tables S1 and S2

## Footnotes

Supplemental information can be found online at https://doi.org/10.1016/j.isci.2021.102710.
