# Supplementary material for: Disease-dependent interaction policies to support health and economic outcomes during the COVID-19 epidemic
Source: iScience. 2021 Jun 10;24(7):102710. doi: 10.1016/j.isci.2021.102710 (PMC8189742; doi:10.1016/j.isci.2021.102710)
Supplement: Document S1. Supplemental procedures, Figures S1–S11, and Tables S1 and S2 [file mmc1.pdf]

iScience, Volume *24*

Supplemental information

Disease-dependent interaction policies to support health and economic outcomes during the COVID-19 epidemic

Guanlin Li, Shashwat Shivam, Michael E. Hochberg, Yorai Wardi, and Joshua S. Weitz

Supplemental Figure 1

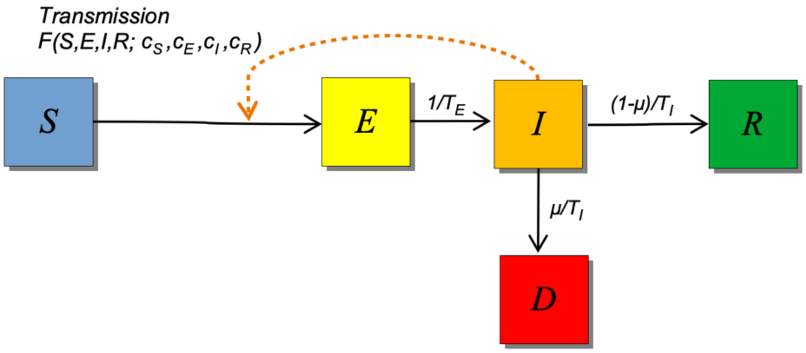

Figure S1. SEIR Model Schematic, related to Figure 1. The interactions between susceptible and infected individuals lead to newly exposed cases. These interactions are modeled by the force of infection *F(S;E;I;R; c)*. The exposed individuals undergo an incubation period (T_E) before the onset of infectiousness. Infectious individuals will either recover (and develop protective immunity) or die after an infectious period (T_I), see model equations in Eq. 2.

Supplemental Figure 2

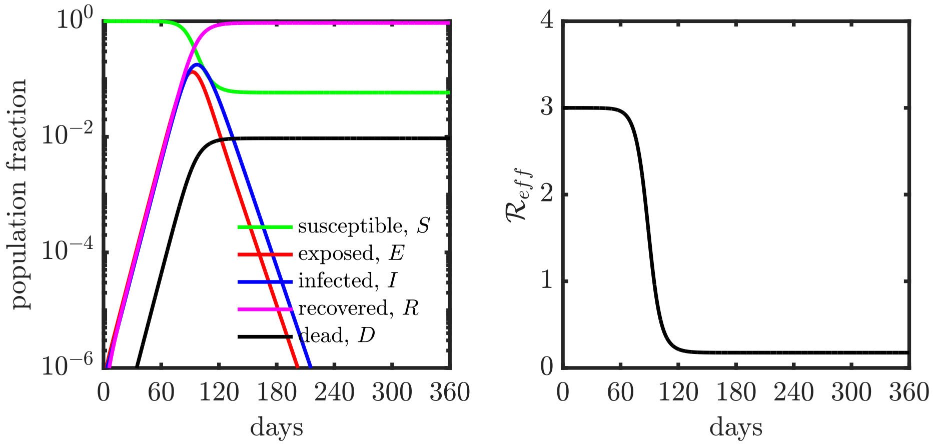

Figure S2. Population dynamics of SEIR model without control, i.e., all c_0's are equal c_B from t_0 to t_f, related to Figure 2. (Left) Population dynamics of SEIR model without control. (Right) Epidemic growth as measured by the effective reproduction number ($\mathcal{R}_{eff}$), the basic reproduction number $\mathcal{R}_0$ is 3.

Supplemental Figure 3

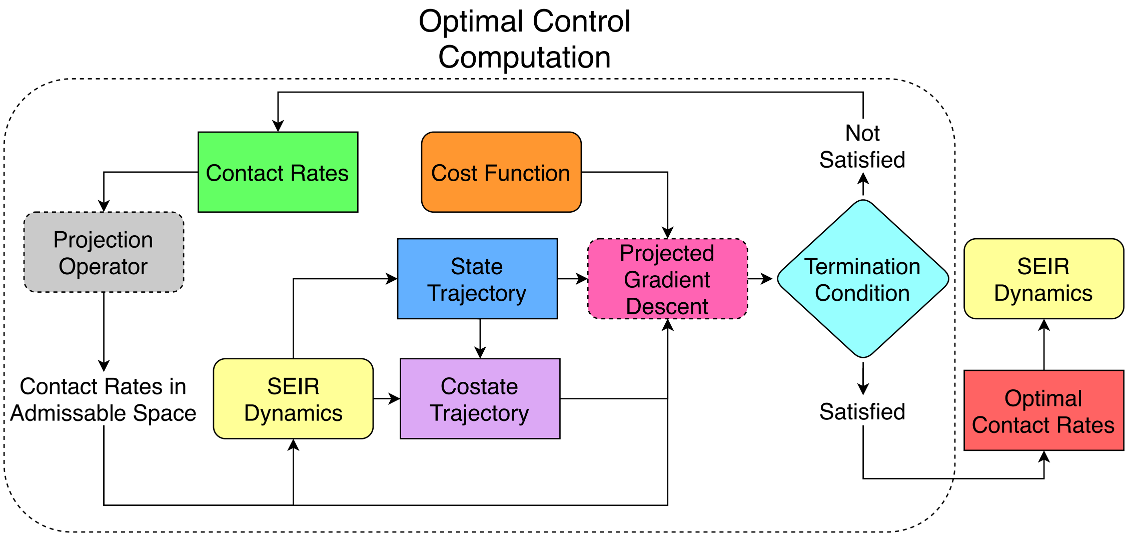

Figure S3. Flowchart of optimal contact rate computation, related to Figure 2. The contact rates are projected into the allowed subspace, and then fed to the system dynamics to generate the state and costate trajectory. Using the cost function provided and the state and costate trajectories, a projected gradient descent algorithm is employed to update the contact rates. If the termination condition is satisfied, these new contact rates are accepted as the optimal contact rates. Otherwise, the contact rates are replaced with the computed values and the loop is rerun.

Supplemental Figure 4

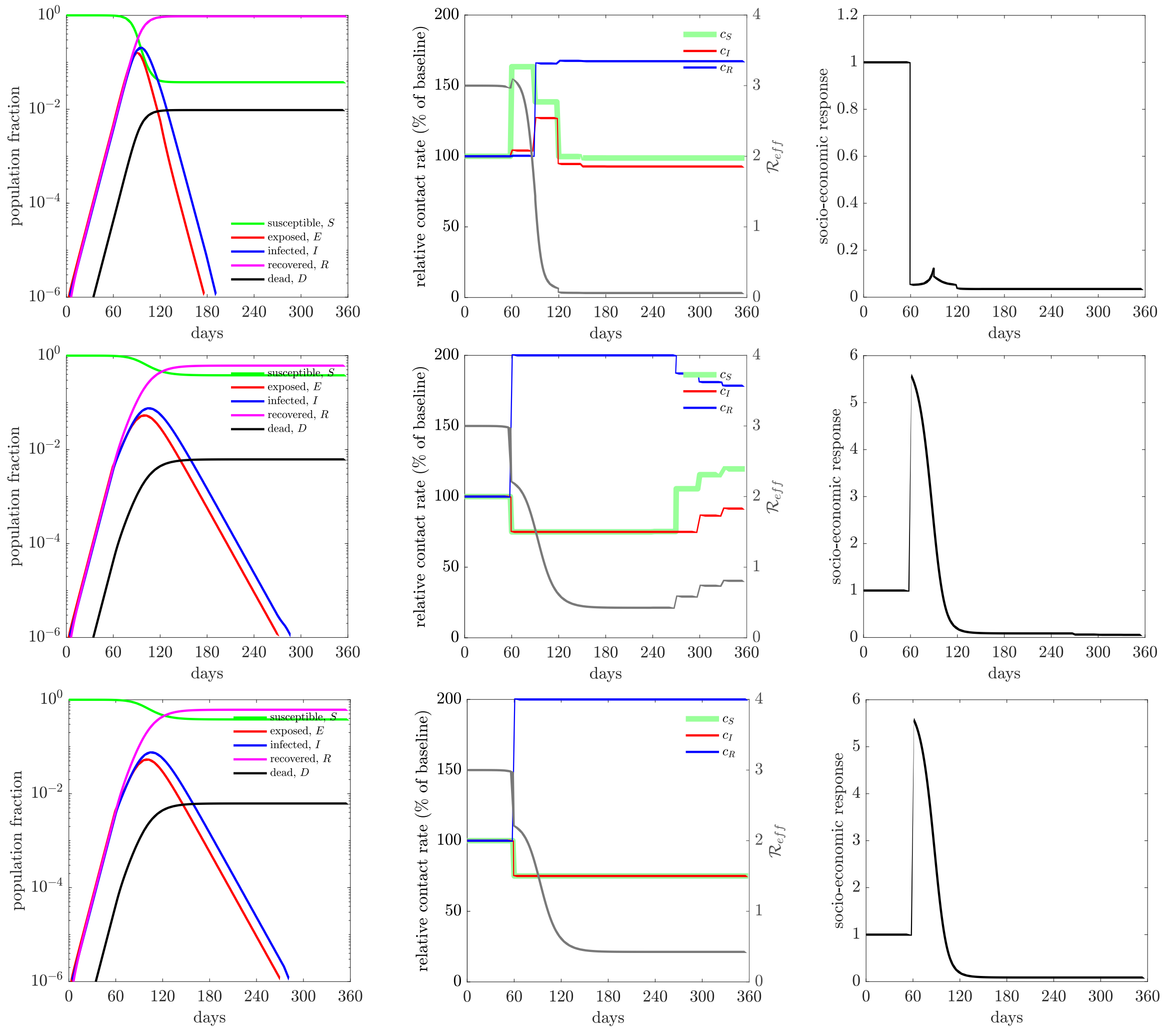

Figure S4. Population dynamics in a SEIR model with controlled contact rate (25% isolation efficiency), related to Figure 3. (Top) The relative importance ratio (ξ) is set as 10^{-4}, the policy (i.e., contact rate) is deployed to primarily minimize socioeconomic costs ($\mathcal{J}_E$). The socioeconomic costs are maintained at low level for all the policy intervention period (see the right panel after 60 days). Final state of the system in terms of population fraction is: $D(t_f) \approx 1\%$; $S(t_f) \approx 4\%$ and $R(t_f) \approx 95\%$, where t_f = 360 days. The fraction of working-days recovered is about 100%. (Middle) The relative importance ratio (ξ) is set as 1, the policy (i.e., contact rate) is deployed to minimize infectious diseases costs ($\mathcal{J}_I$) while keeping the socioeconomic impacts low. The Final state of the system in terms of population fraction is: $D(t_f) \approx 0.6\%$; $S(t_f) \approx 38\%$; and $R(t_f) \approx 61\%$, where t_f = 360 days. The fraction of working-days recovered is about 91%. (Bottom) The relative importance ratio (ξ) is set as 10^4, the policy (i.e., contact rate) is deployed to primarily minimize infectious diseases costs ($\mathcal{J}_I$). The controlled contact rates of susceptible cases (c_S) and infected cases (c_I) are overlapped at minimum contact rate boundary (c_{min}). The Final state of the system in terms of population fraction is: $D(t_f) \approx 0.6\%$; $S(t_f) \approx 38\%$ and $R(t_f) \approx 61\%$, where t_f = 360 days. The fraction of working-days recovered is about 88%. Here, we set c_{min} = $(3/4)c_B$ (i.e., up to 25% isolation efficiency) and c_{max} = $2c_B$ (i.e., up to twice enhanced interactions).

Supplemental Figure 5

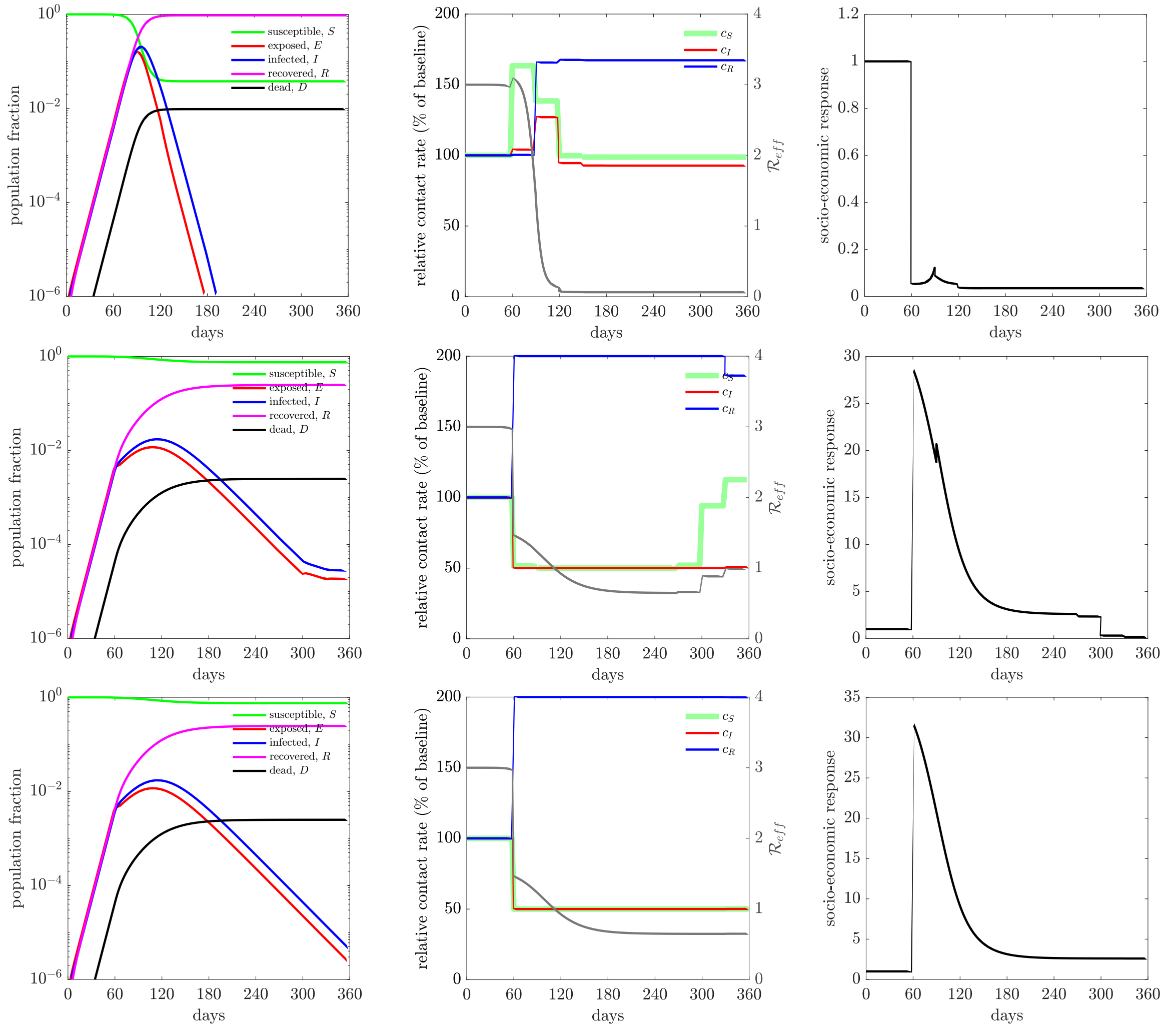

Figure S5. Population dynamics in a SEIR model with controlled contact rate (50% isolation efficiency), related to Figure 3. (Top) The relative importance ratio (ξ) is set as 10^{-4}, the policy (i.e., contact rate) is deployed to primarily minimize socioeconomic costs ($\mathcal{J}_E$). The socioeconomic costs are maintained at low level for all the policy intervention period (see the right panel after 60 days). Final state of the system in terms of population fraction is: $D(t_f) \approx 1\%$; $S(t_f) \approx 3\%$ and $R(t_f) \approx 96\%$, where t_f = 360 days. The fraction of working-days recovered is about 100%. (Middle) The relative importance ratio (ξ) is set as 1, the policy (i.e., contact rate) is deployed to minimize infectious diseases costs ($\mathcal{J}_I$) while keeping the socioeconomic impacts low. For the first half intervention period (from 60 days to 240 days), the controlled contact rates of susceptible cases (c_S) and infected cases (c_I) are overlapped at minimum contact rate boundary (c_{min}). There are huge socioeconomic impacts at beginning of policy intervention period (due to the rapid contact rate reduction of susceptible cases and infected cases, e.g. isolation), the socioeconomic impacts become lower later because of *shielding* (i.e., contact rate of recovered cases is enhanced). Moreover, at the last final 3 months, the socioeconomic costs drop more since the susceptible cases are back to normal (contact rate c_S is increasing). The Final state of the system in terms of population fraction is: $D(t_f) \approx 0.25\%$; $S(t_f) \approx 75\%$; and $R(t_f) \approx 25\%$, where t_f = 360 days. The fraction of working-days recovered is about 66%. (Bottom) The relative importance ratio (ξ) is set as 10^4, the policy (i.e., contact rate) is deployed to primarily minimize infectious diseases costs ($\mathcal{J}_I$). The controlled contact rates of susceptible cases (c_S) and infected cases (c_I) are overlapped at minimum contact rate boundary (c_{min}).

Similar to the intermediate case ($\xi = 1$), large socioeconomic impacts are observed at beginning of policy intervention period. The Final state of the system in terms of population fraction is: $D(t_f) \approx 0.25\%$; $S(t_f) \approx 75\%$ and $R(t_f) \approx 25\%$, where t_f = 360 days. The fraction of working-days recovered is about 88%. Here, we set $c_{min} = (1/2)c_B$ (i.e., up to 50% isolation efficiency) and c_{max} = $2c_B$ (i.e., up to twice enhanced interactions).

Supplemental Figure 6

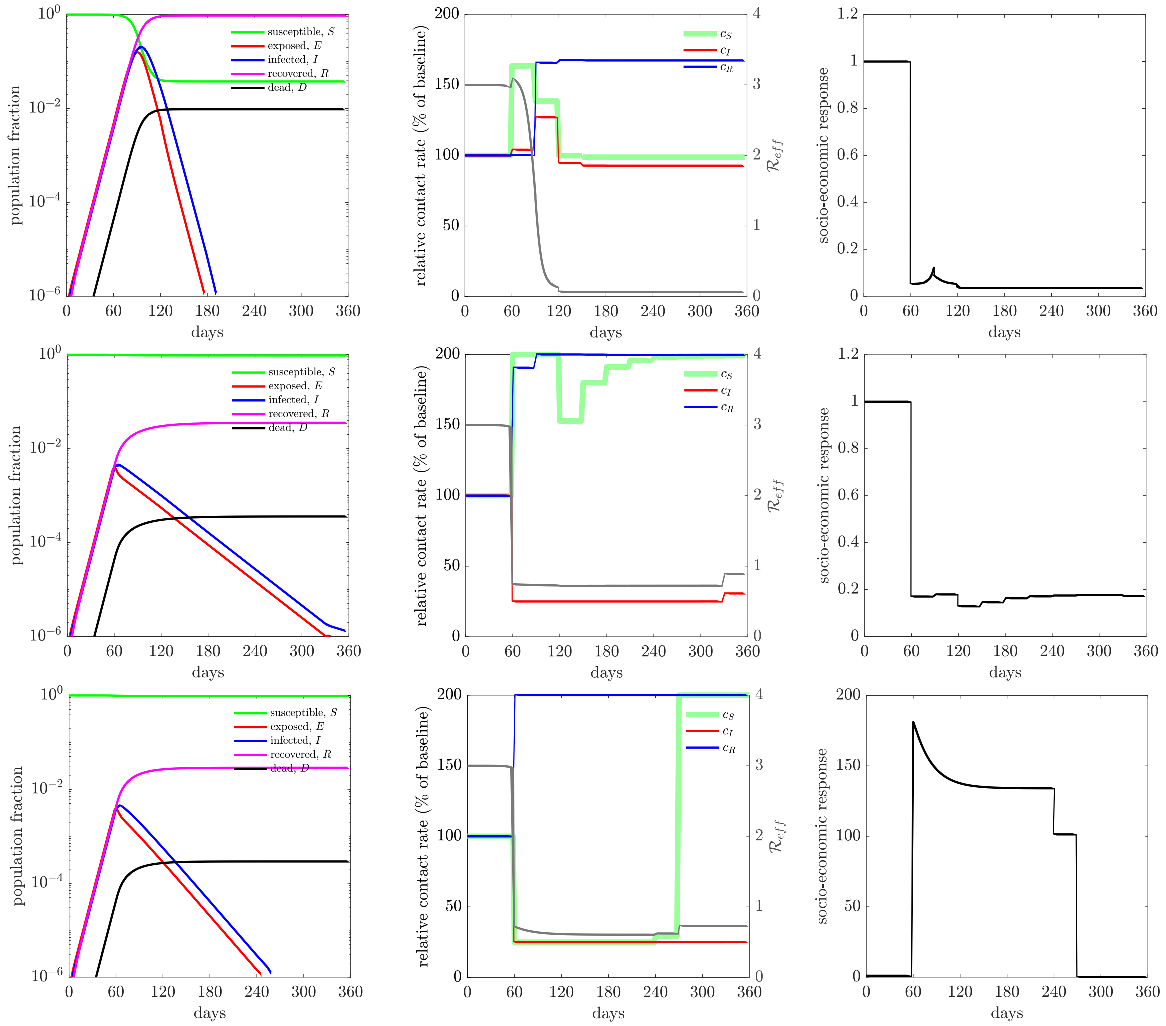

Figure S6. Population dynamics in a SEIR model with controlled contact rate (75% isolation efficiency), related to Figure 3. (Top) The relative importance ratio (ξ) is set as 10^{-4}, the policy (i.e., contact rate) is deployed to primarily minimize socioeconomic costs ($\mathcal{J}_E$). The socioeconomic costs are maintained at low level for all the policy intervention period (see the right panel after 60 days). Final state of the system in terms of population fraction is: $D(t_f) \approx 1\%$; $S(t_f) \approx 4\%$ and $R(t_f) \approx 95\%$, where t_f = 360 days. The fraction of working-days recovered is about 100%. (Middle) The relative importance ratio (ξ) is set as 1, the policy (i.e., contact rate) is deployed to minimize infectious diseases costs ($\mathcal{J}_I$) while keeping the socioeconomic impacts low. Infected individuals are locked down at home for all intervention period while susceptible and recovered individuals are free to go out. The Final state of the system in terms of population fraction is: $D(t_f) \approx 0.04\%$; $S(t_f) \approx 96\%$; and $R(t_f) \approx 4\%$, where t_f = 360 days. The fraction of working-days recovered is about 66%. (Bottom) The relative importance ratio (ξ) is set as 10^4, the policy (i.e., contact rate) is deployed to primarily minimize infectious diseases costs ($\mathcal{J}_I$). The controlled contact rates of susceptible cases (c_S) and infected cases (c_I) are overlapped at minimum contact rate boundary (c_{min}) at beginning, then the susceptibles are free to move later. The Final state of the system in terms of population fraction is: $D(t_f) \approx 0.03\%$; $S(t_f) \approx 97\%$ and $R(t_f) \approx 3\%$, where t_f = 360 days. The fraction of working-days recovered is about 50%. Here, we set $c_{min} = (1/4)c_B$ (i.e., up to 70% isolation efficiency) and c_{max} = $2c_B$ (i.e., up to twice enhanced interactions).

Supplemental Figure 7

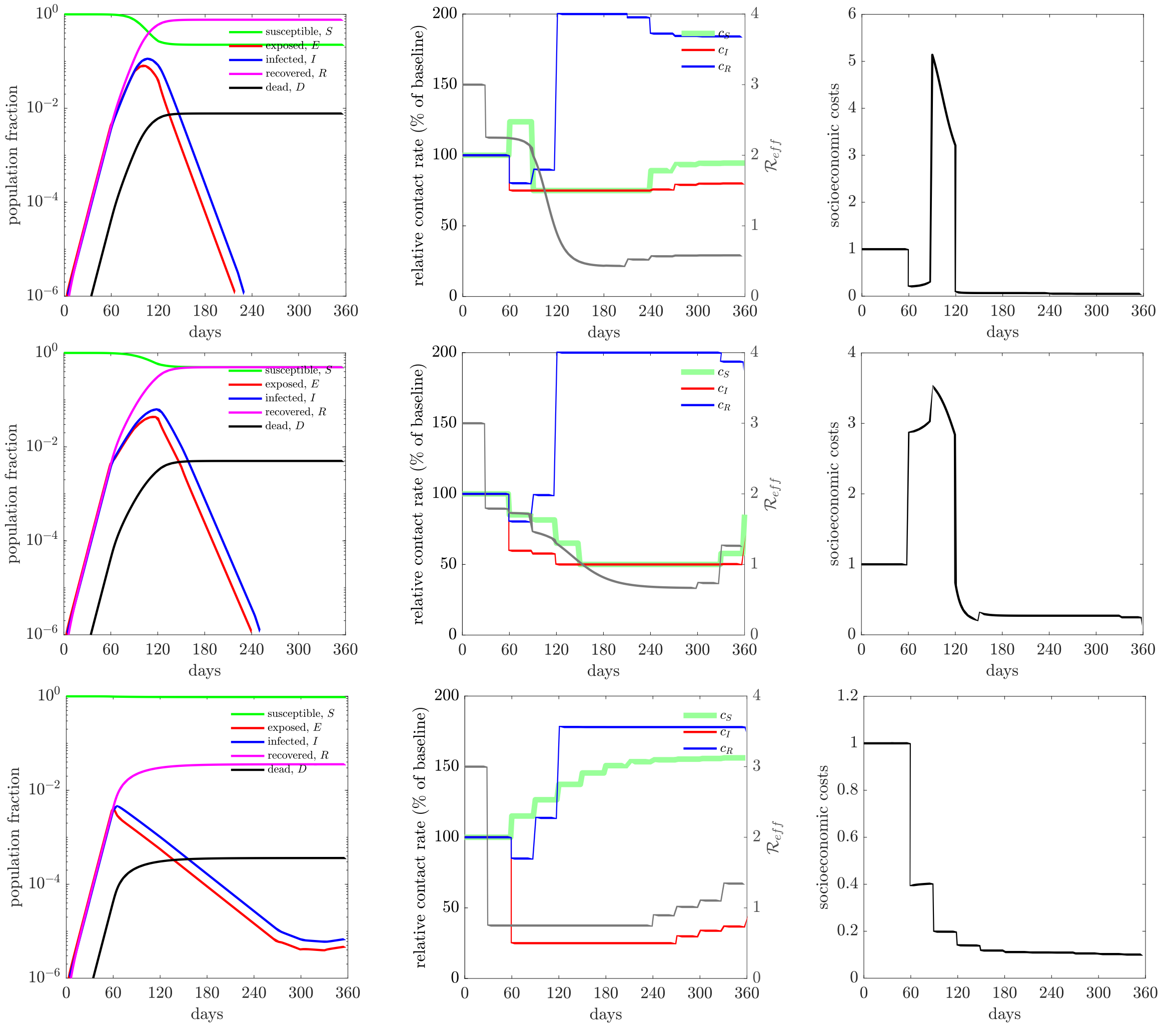

Figure S7. Population dynamics in a SEIR model with mis-timed control policy for various isolation efficiencies: (Top) 25% isolation efficiency; (Middle) 50% isolation efficiency and (Bottom) 75% isolation efficiency, related to Figure 3. The relative importance ratio (ξ) is 1 for all the cases. The optimal control policy is computed for a system one month after an outbreak. Then, we enforce the optimal control policy 30 days later, i.e., at the end of 60 days after the start of the outbreak. The contact rate interventions start at 60 days. The total deaths and working fraction for the delayed system are presented in table S1 for various isolation efficiencies. The optimal control policies and their corresponding dynamics without mistiming are shown in the middle row of Figs. S4, S5, S6.

Supplemental Figure 8

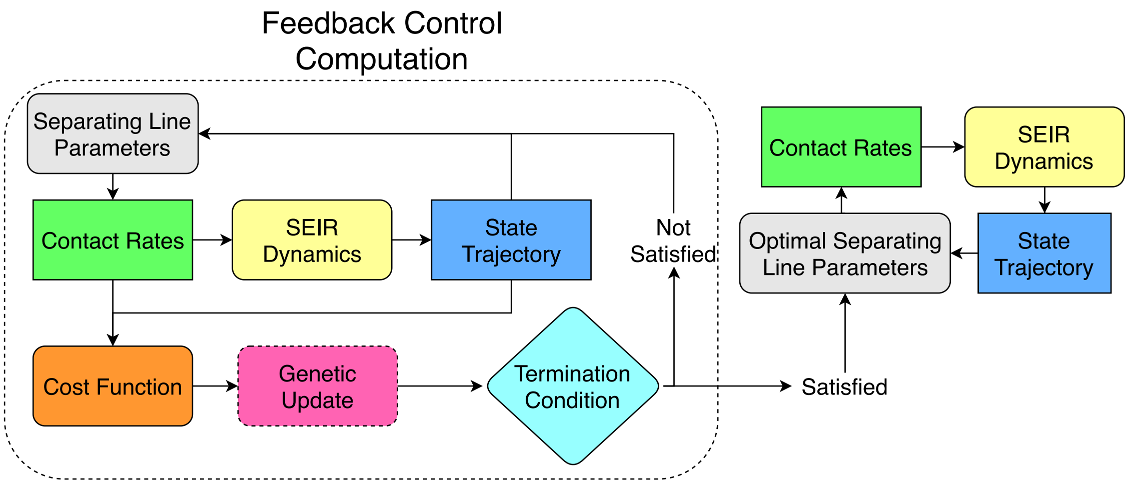

Figure S8. Flowchart for computing state dependent heuristic policy, related to Figure 4. The line dividing the *I*-*R* plane is parameterized and for a particular parameter set, the contact rate is determined solely from the current infected and recovered cases. Starting with an initial parameter guess, the contact rate is computed from the current *I* and *R* values, giving the system's state at the next time instant. After the full state trajectory is computed, the associated cost is calculated and a genetic update step is taken which identifies the next parameters. If the termination condition is satisfied, these parameters are passed on as the optimal parameters which can be used to compute the optimal contact policy. If the termination condition is not satisfied, the line parameters are updated and the loop is run again.

Supplemental Figure 9

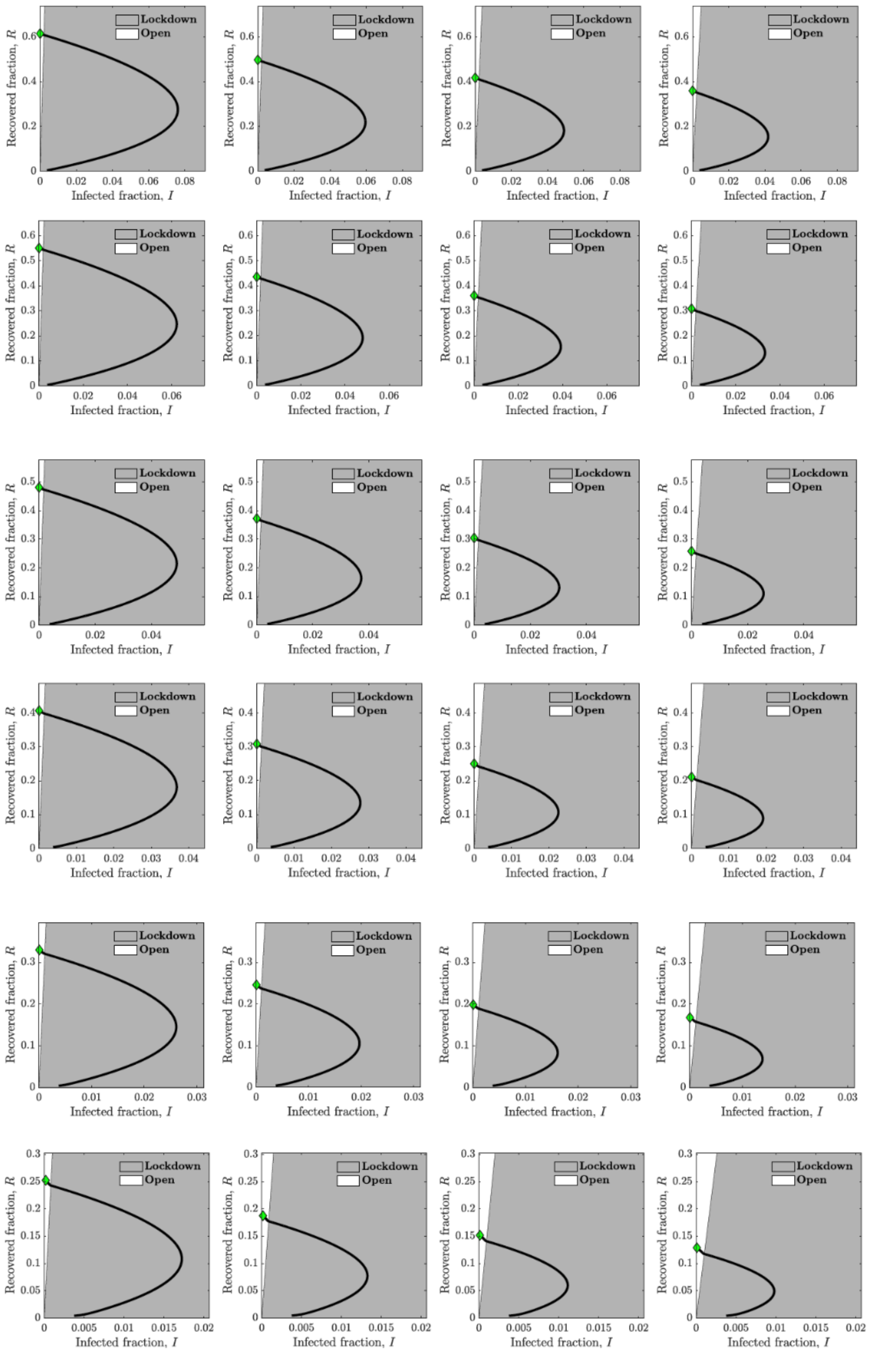

Figure S9. Heuristic state feedback intervention policies varying with isolation efficiency (from 25% to 50%) and shielding level on *I-R* phase plane, related to Figure 4. The isolation efficiency for the first row is 25% and each row increases the efficiency by 5%, which gives the efficiency of the last row as 50%. Next, the 4 columns correspond to shielding levels of 2, 3, 4 and 5 from left to right. In each plot, the grey region specifies the area of the *I-R* plane where a lockdown policy is enforced for the susceptible population and the white region gives the area where the lockdown is lifted i.e. the system is 'open'. The phase trajectory *(I(t); R(t))*, i.e., the black line, crosses from the lockdown to open region when it intersects the dividing line and this crossover happens at most once. The final state of the system is marked by a green diamond.

Supplemental Figure 10

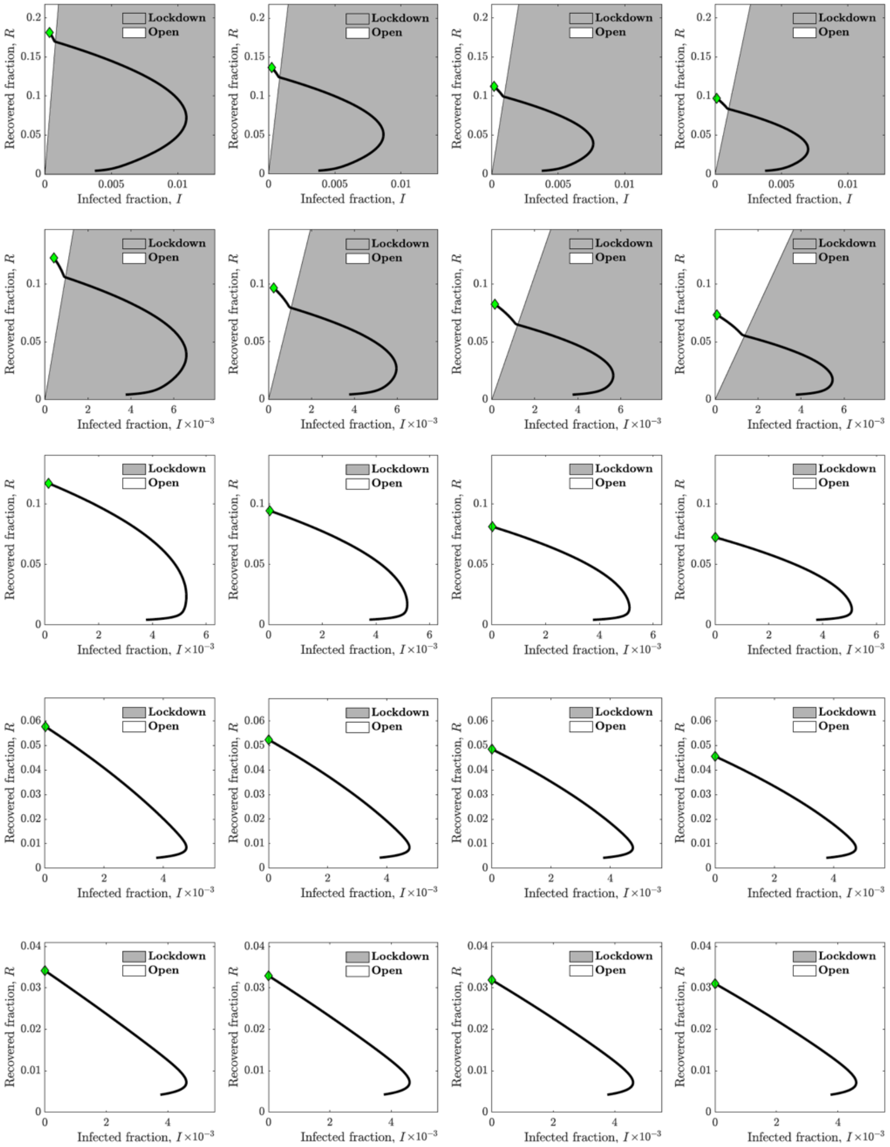

Figure S10. Heuristic state feedback intervention policies varying with isolation efficiency (from 55% to 75%) and shielding level on *I-R* phase plane, related to Figure 4. The isolation efficiency for the first row is 55% and each row increases the efficiency by 5%, which gives the efficiency of the last row as 75%. Next, the 4 columns correspond to shielding levels of 2, 3, 4 and 5 from left to right. In each plot, the grey region specifies the area of the *I-R* plane where a lockdown policy is enforced for the susceptible population and the white region gives the area where the lockdown is lifted i.e. the system is 'open'. The phase trajectory *(I(t); R(t))*, i.e., the black line, crosses from the lockdown to open region when it intersects the dividing line and this crossover happens at most once. The final state of the system is marked by a green diamond.

Supplemental Figure 11

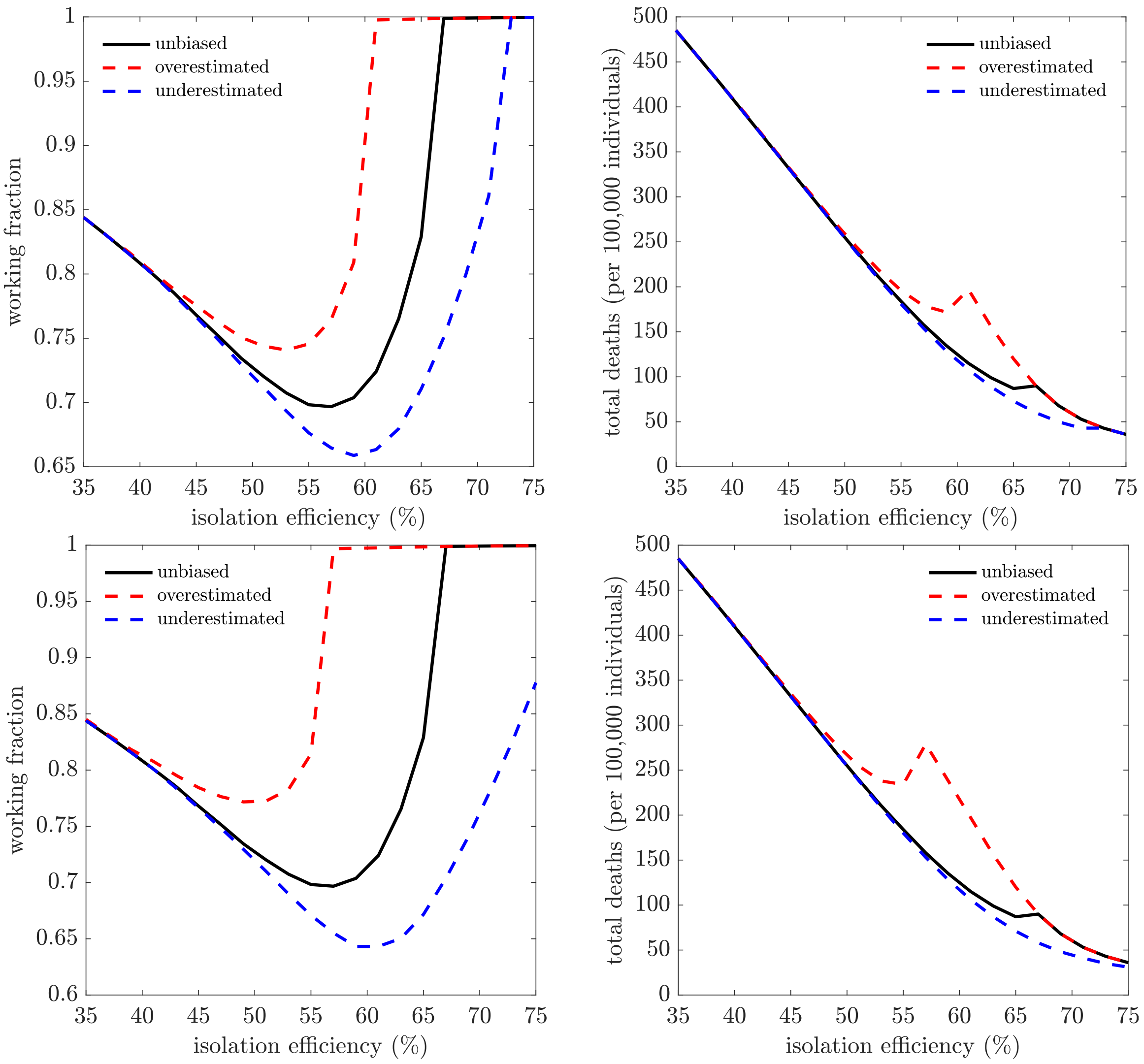

Figure S11. Health and economic outcomes of the feedback control system with estimation error in isolation efficiency, related to Figure 4. The shielding level is fixed at 2, the isolation efficiency varies from 35% to 75% with a 2% increment. (Top) Low uncertainty case. The range of uncertainty is +/- 6%, i.e., $\alpha = 6$. The blue dashed lines represent the working fraction and total death for the system outputs with 6% higher isolation efficiency. The red dashed lines represent the working fraction and total death for the system outputs with 6% lower isolation efficiency. The black lines represent system outputs without mis-specification in isolation efficiency. (Bottom) High uncertainty case. The range of uncertainty is +/-10%, i.e., $\alpha = 10$. The blue dashed lines represent the working fraction and total death for the system outputs with 10% higher isolation efficiency. The red dashed lines are the working fraction and total death for the system outputs with 10% lower isolation efficiency. The black lines represent system outputs without mis-specification on isolation efficiency.

Supplemental Table 1

Parameter	Meaning	Value
η_I	Measure of disease transmission effectiveness	0.1
T_E	Mean incubation period	4 days
T_I	Mean infectious period	6 days
μ	Case fatality ratio of infected cases	0.01
c_B	Baseline potentially infectious contact rate	5/day
$\mathcal{R}_0$	Basic reproduction number	3
r	early exponential growth rate	0.15/day

Table S1. Epidemiological characteristics, related to Figure 2.

Supplemental Table 2

Parameter	Meaning	Value
W_1	Weight of $\mathcal{E}_1$	1
W_2	Weight matrix of $\mathcal{E}_2$	$0.1\mathcal{I}$, $\mathcal{I}$ is identify matrix
W_I	Weight of infection control cost	10^4
W_D	Weight of final deaths cost	10^5
K	Shape parameter in exponential function $\mathcal{E}_1$	7
$\widetilde{w}$	weight of final deaths in heuristic cost	5×10^2

Table S2. Weight regulators and optimization parameters, related to Figures 3 and 4.

Data S1. Methods

1. Model and parameters

The epidemic model analyzed here is an 'SEIR' model (Anderson et al., 1992), including SEIR dynamics, susceptible S, exposed E, infectious I, recovered R , with the total number of fatalities denoted by D. The *force of infection* is the contact rate of infectious individuals multiplied by the probability that the interaction is with a susceptible person multiplied by the probability that the event leads to an infection. Let c_S, c_E, c_I and c_R equal the contact rate of S, E, I, and R individuals respectively. Although such different rates imply the existence of an implicit contact matrix, we are only focused on infection dynamics associated with a single epidemiological birth state, such that the force of infection can be represented by a single term, $F(S,E,I,R;c)$. The force of infection is

$$F(S,E,I,R;c) = \eta_I c_I \left(\frac{c_S S}{c_S S + c_E E + c_I I + c_R R}\right),$$

(1)

where η_I is the measure of disease transmission effectiveness from I class to S class (Ngonghala CN, et al., 2020). Suppose all the contact rates are equal to a baseline contact rate c_B. Assuming relatively few fatalities per-capita, then the total population is approximately constant at N, such that the force of infection can be approximated as $F \approx \eta_I c_B (S/N)$, which recovers the standard SEIR model by defining $\beta = \eta c_B$ as *infection rate*. In the generalized case, the model is given by the following system of nonlinear differential equations

$$\begin{aligned}
\frac{dS}{dt} &= -F(S,E,I,R;c)I \\
\frac{dE}{dt} &= F(S,E,I,R;c)I - \frac{1}{T_E}E \\
\frac{dI}{dt} &= \frac{1}{T_E}E - \frac{1}{T_I}I \\
\frac{dR}{dt} &= (1-\mu)\frac{1}{T_I}I \\
\frac{dD}{dt} &= \mu \frac{1}{T_I}I,
\end{aligned}$$

(2)

where $F(S,E,I,R;c)$ is the force of infection given in Eq. 1, and T_I is the infectious period, T_E is the incubation period, μ is the case fatality ratio for infected individuals, see SEIR model schematic in Fig. S1.

At the start of an outbreak ($t = 0$), the contact rate of each individual is c_B. The basic reproduction number $\mathcal{R}_0$ of this compartmental model is

$$\mathcal{R}_0 = \overbrace{T_I}^{\text{average infectious time}} \times \overbrace{\eta_I c_B}^{\text{infection rate}} .$$

(3)

The initial exponential growth rate (r) of the epidemic spread is given by the largest eigenvalue of the Jacobian matrix evaluated around the disease-free equilibrium (DFE). Linearizing system (2) about the DFE, the Jacobian matrix of infected subsystem reads

$$J_0 = \begin{bmatrix} -1/T_E & \eta_I c_B \\ 1/T_E & -1/T_I \end{bmatrix}.$$

(4)

Then, the largest eigenvalue of J_0 (*i.e.,* early exponential growth rate r) is

$$r = \frac{1}{2}\left[-\left(\frac{1}{T_E} + \frac{1}{T_I}\right) + \sqrt{\left(\frac{1}{T_E} - \frac{1}{T_I}\right)^2 + 4\frac{\eta_I c_B}{T_E}}\right].$$

(5)

The assumed model parameters used in the model are shown in table S1.

Initial conditions and contact rate

The model assumes an initial total population of $1{,}000{,}000$ (one million) denoted as N_0. An initial outbreak is seeded in this population given one infected individual. The simulation is run for two months (60 days) with fixed baseline contact rate (all c's are equal to c_B) - we use this time point (which we denote time t_0 in our simulations) as the time at which intervention policies might be applied. The individual behaviors (or activities) are quantified by contact rates, $c_S(t),\ c_E(t),\ c_I(t)$ and $c_R(t)$, thus the control of contact rates can be mapped to intervention strategies. For examples, when c_S goes down that means shelter-in-place, when c_E or c_I goes down that can mean quarantine and isolation. When there is no intervention during the epidemic, the population dynamics of SEIR model is shown in Fig. S2. At the time intervention starts (60 days), the disease spreads through the population without any intervention leading to incidence 19 (per 10, 000), prevalence 0.4%, and a cumulative infection level of 0.8%.

We denote $c(t) = (c_S(t), c_E(t), c_I(t), c_R(t))^T$ as the contact rate vector at time t, where $(\cdot)^T$ denotes a matrix transpose. The effective reproduction number $\mathcal{R}_{eff}(t)$ is

$$\mathcal{R}_{eff}(t) = \overbrace{\bar{T}_I}^{\text{average infectious time}} \times \overbrace{\eta_I(t)c_I(t)\frac{c_S(t)S(t)}{Q(t)}}^{\text{infection rate in susceptible population}},$$

(6)

where $Q(t) = c_S(t)S(t) + c_E(t)E(t) + c_I(t)I(t) + c_R(t)R(t)$.

2. Optimal control formulation

Here, we aim to optimally deploy public health control strategies (via control of the contact rates) that minimizes deaths during the outbreak while keeping the socioeconomic costs low. The baseline average of contacts at time t, $Q(t)$, equals $c_B N(t)$, where c_B is the baseline contact rate and $N(t) = S(t) + E(t) + I(t) + R(t)$ is the total number of alive individuals The socioeconomic costs may result from two consequences of contact rate interventions: (1) loss of essential connections; (2) shifting of roles relative to baseline, as quantified by variation in contact rates by testing status. Given the intervention policy $c(t)$ at time t, we quantify the socioeconomic costs as $\mathcal{E}_1 + \mathcal{E}_2$, where $\mathcal{E}_1$ and $\mathcal{E}_2$ may take form of

$$\begin{aligned}
\mathcal{E}_1(Q(t); W_1) &= W_1 \exp\left(K\left(\frac{c_B N(t) - Q(t)}{c_B N(t)}\right)\right) \\
\mathcal{E}_2(c(t); W_2) &= \frac{1}{2}\sum_{i\in\{S,E,I,R\}} w_i\left(\frac{c_B - c_i(t)}{c_B}\right)^2 \\
&= \frac{1}{2}\left(\frac{c_B - c(t)}{c_B}\right)^T W_2 \left(\frac{c_B - c(t)}{c_B}\right),
\end{aligned}$$

(7)

where $W_1 \geq 0$, $W_2 = \mathrm{diag}(w_S, w_E, w_I, w_R) \succ 0$ are weight parameters, and K is a constant shape parameter for the exponential function. In the absence of any strategy, there are few deaths. This would mean that $N(t) \approx N_0$. For the sake of simplicity, $\mathcal{E}_1$ is approximated as $W_1 \exp(K(c_B N_0 - Q(t))/c_B N_0)$. For every t, we restrict the contact rate vector $c(t) \in [c_{min}, c_{max}]^4$, where c_{min} and c_{max} are the minimum contact rate and maximum contact rate. The set $C = [c_{min}, c_{max}]^4$ is a convex and compact set in $\mathbb{R}^4$. We define the space of *admissible controls*, denoted by $\mathcal{A}$, as the set of Lebesgue-measurable functions $c: [t_0, t_f] \to C$. The (dimensionless) cost functional is written in the following Bolza form,

$$\mathcal{J}(c) = \overbrace{W_D \frac{D(t_f)}{N_0}}^{\text{costs of deaths}} + \int_{t_0}^{t_f} \overbrace{\mathcal{E}_1(Q(t); W_1) + \mathcal{E}_2(c(t); W_2)}^{\text{socioeconomic costs}} + \overbrace{W_I \frac{I(t)}{N_0}}^{\text{infectious case costs}} \quad \mathrm{dt},$$

(8)

where $W_I \geq 0$ and $W_D \geq 0$ are weight regulators. The optimal control problem is

$$\min \{\mathcal{J}(c) | c \in \mathcal{A}\}.$$

(9)

The weight parameters used in Eq. 8 are shown in table S2, these weight parameters are set to balance the scale of different cost components in Eq. 8.

However, frequent and continuous updates bring in practical issues in terms of (1) establishing how subtle changes in contact rates actually translate into applicable changes in individual behaviors (*i.e.*, a continuous strategy can not be practically implemented), and (2) individual fatigue (and reduced adherence to proposed measures) due to frequent behavioral adjustments (*i.e.*, frequent update is also impractical). To address these concerns, we need to modify the space of controls $\mathcal{A}$.

To begin with, we fix the full time horizon of control as $[t_0, t_f]$. For example, t_0 is fixed to 60 days after outbreak (see Sect. 1) and t_f to 360 days after outbreak, where we set the timing of the outbreak to 0. We only consider an intervention policy which is updated a finite number of times in the interval $[t_0, t_f]$. These epochs are denoted by $t_0 < t_1 < t_2 < \cdots < t_M = t_f$. Let $\mathcal{O}$ denote the corresponding partition of $[t_0, t_f]$, and denote by $\mathcal{F}(\mathcal{O})$ the space of functions $c: [t_0, t_f] \to \mathbb{R}^4$ which have constant values in the intervals $[t_{m-1}, t_m)$, $m = 1,2, \ldots$. The resulting optimal control problem is

$$\min \{\mathcal{J}(c) | c \in \mathcal{A} \cap \mathcal{F}(\mathcal{O})\}.$$

(10)

In most cases, the exposed individuals are unaware of their infection status, so it's impractical to control their behaviors separately from the susceptible individuals. Hence, we view them as susceptibles for the purpose of policy setting and set $c_E(t) = c_S(t)\ \forall t \in [t_0, t_f]$. In doing so, the contact rate vector $c(t)$ actually is a three dimensional vector consisting of $c_S(t)$, $c_I(t)$ and $c_R(t)$

.

3. Optimization algorithm

We follow the framework presented in Polak (Plolak., 1997) for computational optimal control based on optimization on Hilbert spaces. We first discuss the conceptual algorithm in an abstract setting of infinite-dimensional spaces, and then provide approximation details in the sequel.

Let $L_2^n[t_0, t_f]$ denote the space of equivalence classes of square integrable functions from $[t_0, t_f]$ into $\mathbb{R}^n$ ($n = 3$ in our case). We use the notation $\|\cdot\|$ and $\langle \cdot,\cdot \rangle$ for the norm and scalar product in Euclidean space (e.g. $\mathbb{R}^n$), and use $\|.\|_{\mathcal{H}}$ and $\langle .,. \rangle_{\mathcal{H}}$ for the norm and scalar product in a Hilbert space $\mathcal{H}$. For example,

given $u, v \in L_2^n[t_0, t_f]$ (a Hilbert space), the norm $\|.\|_2$ and scalar product $\langle .,. \rangle_2$ are: $\|u\|_2 = \sqrt{\int_{t_0}^{t_f} \|u(t)\|^2 \, \mathrm{dt}}$; $\langle u, v \rangle_2 = \int_{t_0}^{t_f} \langle u(t), v(t) \rangle \mathrm{dt}$.

In the optimal control problem under consideration, we define the state variable of the system, $x \in \mathbb{R}^5$, by $x = (S, E, I, R, D)^T$. We also define the control variable, $c \in \mathbb{R}^3$, by $c = (c_S, c_I, c_R)^T$. The dynamic equation of the system, Eq. 2, can be rewritten in a compact form:

$$\dot{x}(t) = f(x(t), c(t)),$$

(11)

where dot represents differentiation with respect to time t, and $f(x(t), c(t))$ is the right hand side (RHS) of equations 2. The cost functional defined by Eq. 8 can be written succintly as

$$\mathcal{J}(c) = \phi(x(t_f)) + \int_{t_0}^{t_f} L(x(t), c(t)) \, \mathrm{dt},$$

(12)

where $L(x(t), c(t)) := \mathcal{E}_1(Q(t); W_1) + \mathcal{E}_2(c(t); W_2) + W_I(I(t)/N_0)$ and $\phi(x(t_f)) = W_D(D(t_f)/N_0)$. The costate (adjoint) variable, $\lambda(t) \in \mathbb{R}^5$, is defined by the following equation,

$$\dot{\lambda}(t) = -\left(\frac{\partial f}{\partial x}(x(t), c(t))\right)^T \lambda(t) - \left(\frac{\partial L}{\partial x}(x(t), c(t))\right)^T, \qquad \lambda(t_f) = \nabla_x \phi(x(t_f)).$$

(13)

Next, recall the optimal control problem defined by Eq. 10, where the cost functional (performance integral) $\mathcal{J}: \mathcal{A} \to \mathbb{R}$ is defined by (12). We will propose a gradient-descent algorithm for solving this problem, where the gradient $\nabla \mathcal{J}(c(t))$ is given by the following equation,

$$\nabla \mathcal{J}(c(t)) = \left(\frac{\partial f}{\partial c}(x(t), c(t))\right)^T \lambda(t) + \left(\frac{\partial L}{\partial c}(x(t), c(t))\right)^T;$$

(14)

See (Plolak., 1997, Kirk., 2004, Liberzon., 2011).

The principal elements of most gradient-descent algorithms (including the one described here) are the descent direction and the stepsize. For our problem, the descent direction is based on $-\nabla \mathcal{J}(c(t))$; in fact, it is the projection of $c(t) - \nabla \mathcal{J}(c(t))$ into the constraint-set $C = [c_{min}, c_{max}]^3$ as defined in Sect. 1. The stepsize that we use is the Armijo stepsize (see Plolak., 1997) described in the sequel, which gives an approximate line minimization of $\mathcal{J}$ in the computed descent direction from the control $c(t)$.

Consider first the conceptual algorithm. In its formal presentation by Algorithm 1, below, we use the term "compute" in a conceptual sense, since the "computations" refer to elements in the Hilbert space $L_2^n[t_0, t_f]$. Also, we use the notation $\mathcal{P}_{\mathcal{A}}$ to mean the projection from $L_2^n[t_0, t_f]$ into the space $\mathcal{A}$.

Algorithm 1: Conceptual.

Given a control iteration $c^{(i)} \in \mathcal{A}$, compute from it the next iteration, $c^{(i+1)} \in \mathcal{A}$, as follows.

Parameters: Constants $\alpha \in (0,1)$ and $\beta \in (0,1)$. (*These parameters play a role in the computation of the step size.*)

1. Compute the state trajectory $x(t)$ and costate trajectory $\lambda(t)$ by Eqs. 11 and 13, respectively.

2. Compute $\nabla\mathcal{J}(c(t))$ by Eq. 14.

3. (*Here we define, for all* $y \geq 0$, $h^{(i)}(y) = \mathcal{P}_{\mathcal{A}}(c^{(i)} - y\nabla\mathcal{J}(c^{(i)}))$). Compute $\ell(i) > 0$ defined as follows,

$$\ell(i) = \min\{\ell = 0,1,\ldots \mid \mathcal{J}(h^{(i)}(\beta^{\ell})) - \mathcal{J}(c^{(i)}) \leq -\alpha\langle \nabla\mathcal{J}(c^{(i)}), c^{(i)} - h^{(i)}(\beta^{\ell})\rangle_2\}.$$

4. Set $\gamma(i) = \beta^{\ell(i)}$, and set

$$c^{(i+1)} = \mathcal{P}_{\mathcal{A}}(c^{(i)} - \gamma(i)\nabla\mathcal{J}(c^{(i)})).$$

We remark that $\mathcal{P}_{\mathcal{A}}(c^{(i)} - \gamma(i)\nabla\mathcal{J}(c^{(i)})) - c^{(i)}$ is descent direction for $\mathcal{J}$ from $c^{(i)}$, and $\gamma(i)$, the Armijo stepsize, provides an approximate-line minimization in that direction.

The main modification of Algorithm 1 towards implementation is in numerical solutions of the state equation and costate equation. Furthermore, the time-interval $[t_0, t_f]$ has to be discretized by a suitable grid in order to adequately represent various time-dependent functions such as $c^{(i)}(t)$ and $\nabla\mathcal{J}(c(t))$. All of this can be achieved by a common grid, $\mathcal{G}_0 := \{t_0, t_1, \ldots, t_N\} \subset [t_0, t_f]$. Furthermore, the projection $\mathcal{P}_{\mathcal{A}}(c^{(i)} - \beta^{\ell}\nabla\mathcal{J}(c^{(i)}))$ in Step 3 of Algorithm 1 can be computed as follows: For a given grid-point $t_j \in \mathcal{G}_0$, project the point $c^{(i)}(t_j) - \beta^{\ell}\nabla\mathcal{J}(c^{(i)})(t_j) \in \mathbb{R}^3$ into the set C, where $C = [c_{min}, c_{max}]^3 \subset \mathbb{R}^3$. This can be done by a simple co-ordinate projection since C is a box. Denote the result of this pointwise projection by $\mathcal{P}_C(c^{(i)}(t_j) - \beta^{\ell}\nabla\mathcal{J}(c^{(i)})(t_j)) \in C$, and observe that the function comprised of a zero-order or first-order interpolation of these points can serve to approximate the functional projection $\mathcal{P}_{\mathcal{A}}(c^{(i)} - \beta^{\ell}\nabla\mathcal{J}(c^{(i)}))$.

These modifications of the conceptual algorithm give an implementable version for it. The resulting implementable algorithm falls in Polak's framework of numerical optimal control (Plolak., 1997) which includes results pertaining to asymptotic convergence.

As noted, the control $c(t)$ must be piecewise constant and maintain constant values for substantial periods. At the same time, the state trajectory typically is continuous and not piecewise constant. Therefore, it is natural to maintain the present framework of continuous-time optimal control and not to consider the problem in the setting of discrete-time systems. To do that, we have to consider a form of projection of the control functions, $c(t)$, into the space of piecewise-constant functions. We next describe the specific way we did that in our simulations.

Consider an increasing set of points $\{t_0 = t_0 < t_1 < t_2 < \ldots < t_M = t_f\}$, and the corresponding partition of the time-interval $[t_0, t_f)$ by the subintervals $[t_{m-1}, t_m)$, $m = 1, \ldots, M$. Denote the interval $[t_{m-1}, t_m)$, $m = 1, \ldots, M$, by Δ_m; right-close the last interval to be $\Delta_M = [t_{M-1}, t_M]$, and denote the partition by $\mathcal{O}$. Let $\psi_m(t)$, $m = 1, \ldots, M$ denote the indicator function of the subinterval ψ_m, namely $\psi_m(t) = 1$ for $t \in \psi_m$ and $\psi_m(t) = 0$ otherwise. A continuous-time signal can be approximated over an interval by a constant equal to its average value over that interval. Given a continuous-time policy $c: [t_0, t_f] \to \mathbb{R}^3$, the piecewise constant reconstruction is

$$\tilde{c}\,(t) = \sum_{m=1}^{M-1} \theta_m\, \psi_m(t), \quad \theta_m = \frac{1}{|\Delta_m|}\int_{t_m}^{t_{m+1}} c\,(s)\ \mathrm{ds},$$

(15)

here $\theta_m \in \mathbb{R}^3$. Let $\mathcal{V}_O$ denotes the operator comprising this piecewise- constant reconstruction procedure. Then Eq. 15 can be written as $\tilde{c} = \mathcal{V}_O(c)$. Now we modify the algorithm by replacing the projection operator $\mathcal{P}_{\mathcal{A}}$ in Step 3 by the operator $\mathcal{V}_O \circ \mathcal{P}_{\mathcal{A}}$, comprised of $\mathcal{P}_{\mathcal{A}}$ followed by $\mathcal{V}_O$.

In summary, the implementable algorithm that we use consists of the following modification of Algorithm 1:

1. Compute $x(t)$ and $\lambda(t)$ by numerical integrations using the forward-Euler method.
2. Compute $\nabla\mathcal{J}(c(t))$ only at the points t_j on the grid $\mathcal{G}_0$.
3. Redefine $h^{(i)}(y)$ as $h^{(i)}(y) = \mathcal{V}_O \circ \mathcal{P}_{\mathcal{A}}(c^{(i)} - y\nabla\mathcal{J}(c^{(i)}))$.
4. Replace $\mathcal{P}_{\mathcal{A}}$ by $\mathcal{V}_O \circ \mathcal{P}_{\mathcal{A}}$.

A flow chart of the algorithm is depicted in Fig. S3.

Simulation details

The model assumes an initial total population of $1{,}000{,}000$ (one million) denoted as N_0. An initial outbreak is seeded in this population given one infected individual. The simulation is run for two months (60 days) with fixed baseline contact rate (all c's are equal to c_B) - we use this time point (which we denote time t_0 in our simulations) as the time at which intervention policies might be applied: we have $c(t) = c_B \; \forall t \in [0, t_0]$, and we set the initial state to $x_0 = x(t_0)$.

For the algorithm, we set $\alpha = 0.1$ and $\beta = 0.5$, the grid's time increments to 0.05, $\Delta_m = 30$ days, $t_0 = 60$ and $t_f = 360$ days. For the uncontrolled part of the simulation we take $c(t) = c_B$ for all $t \in [0, t_0]$. The term $\Theta(i) := |\mathcal{J}(c^{(i+1)}) - \mathcal{J}(c^{(i)})|/\mathcal{J}(c^{(i)})$ acts as a convergence indicator for stopping the algorithm. The algorithm is terminated whenever $\Theta(i) \leq 10^{-6}$.

4. Optimised time-dependent contact rate

One way of estimating the economic impact is measuring the *fraction of working-days recovered*, *i.e.,* how many days are people working. We assume that if a person has a contact rate less than the base contact rate (c_B), it implies a proportional reduction of in-person contacts that have a direct economic benefit. For example, for a susceptible individual, the working fraction at day t can be computed as $c_S^*(t) = \min\{1, c_S(t)/c_B\}$, where c_B is the baseline contacting rate. This ensures that contact rates above the baseline does not correspond to an increase in hours worked. For a given contact rate policy $c = (c_S, c_E, c_I, c_R)^T$, the working days restored in the period $[t_0, t_f]$ is

$$\Psi_{restored} = \int_{t_0}^{t_f} c_S^*(t)S(t) + c_E^*(t)E(t) + c_I^*(t)I(t) + c_R^*(t)R(t)\,\mathrm{dt},$$

(16)

where $c_k^*(t) = \min\{1, c_k(t)/c_B\}$, $k \in \{S, E, I, R\}$, is the working fraction of an individual in subpopulation class k at day t. When there is no policy intervention, the contact rate of each individual is c_B for all time, the working days will be maximized, which can be written as

$$\Psi_{max} = \int_{t_0}^{t_f} S(t) + E(t) + I(t) + R(t)\,\mathrm{dt}.$$

(17)

Then, the fraction of working days restored can be written as

$$\Psi(c) = \frac{\Psi_{restored}}{\Psi_{max}}.$$

(18)

Economic costs are proportional to $1 - \Psi(c)$; such that economic impacts of policies are minimized as $\Psi(c)$ approaches 1.

Varying the relative importance of the illness-related cost vs. socioeconomic costs

The relative importance given to the socioeconomic impact of the epidemic and the cost associated with deaths and infection spread is critical to design intervention policies. From Eq. 8, we note that the cost function $\mathcal{J}$ can be decomposed into the two parts, socioeconomic costs ($\mathcal{J}_E$) and costs of infectious diseases ($\mathcal{J}_I$), $\mathcal{J} = \mathcal{J}_E + \mathcal{J}_I$, where

$$\begin{aligned} \mathcal{J}_E &= \int_{t_0}^{t_f} \mathcal{E}_1\,(Q(t); W_1) + \mathcal{E}_2(c(t); W_2)\ \mathrm{dt} \\ \mathcal{J}_I &= W_D \frac{D(t_f)}{N_0} + \int_{t_0}^{t_f} W_I \frac{I(t)}{N_0}\ \mathrm{dt}. \end{aligned}$$

(19)

In order to explore how to manage and control disease outbreak with low socioeconomic costs, we need to balance the importance of these two seemingly conflicting objectives. To this end, we define a new cost function where the relative importance of $\mathcal{J}_E$ and $\mathcal{J}_I$ can be parameterized. With this parameter, optimal policy decisions can be made based on the relative importance assigned to the socioeconomic effect and public health impacts. Define the relative weight ratio as ξ and the altered cost function is $\mathcal{J}(c;\xi) = \mathcal{J}_E + \xi\mathcal{J}_I$. As ξ increases, the relative importance of costs associated with death and spread of infection are increased vis-a-vis the socioeconomic impact and vice versa. The optimal intervention policies with different relative importance ratios (ξ = $10^{-4}, 1$ and 10^{4}) vary with isolation efficiencies ($25\%,\ 50\%,\ 75\%$) are shown in Figs. S4, S5, S6.

For example of balance case ($\xi = 1$) in the case of 50% isolation efficiency, Fig. S5 (middle) shows that the way of managing the epidemic while considering the socioeconomic impact is to isolate all the infected cases and enhance the interactions of recovered cases (*i.e., shielding*, Weitz et al., 2020), the susceptible cases are stay home first and slowly go back to normal towards the end of intervention period. Using the optimized intervention policy (*i.e.,* optimal contact rate), the reduction in final deaths ($D(t_f)$) is substantial ($\sim 75\%$). The fraction of working-days recovered is about $\sim 66\%$. The results suggest advantages of isolation of infected cases and shielding enhancement of recovered cases.

5. Inferring state-dependent heuristic policies from optimal control solutions

In the previous sections, an optimal policy based on contact rates of different sub-populations was derived. However, as is often the case with optimal control solutions, these policies are open-loop, that is, once computed, they are prescribed to the system without feedback. Therefore, open-loop policies can have large sensitivities to modeling errors and signal-delays in the loop, especially if the system is unstable, as is the case with dynamic models of epidemics near the disease-free equilibrium at the onset of an outbreak. To address this, we design a feedback law for optimizing a ‘reasonable’ performance metric which, though different from the one of the optimal control problem, gives similar respective measures of two important performance metrics: total mortality ($D(t_f)$), and working fractions (Ψ). In Sect. 6 we verify this point by comparative simulations of the optimal control solution and the feedback control system, and not surprisingly, the sensitivity of the former with respect to a one-cycle (30-days) delay is much larger than that of the latter.

The optimal control solutions reveal a general structure that guides us in the choice of the feedback control law. The patterns observed in the computed optimal-policy solutions (see Figs. S4, S5, S6) suggest that the contact rates for the infected cases should be the smallest possible while the recovered

population should be used as shields. Further, they display switch overs between the low and high contact rates for the susceptible populations, and these tend to occur towards the end of the lock-down period in a way that depends on the numbers of infected and recovered individuals.

To find the state feedback policy, the I-R (Infected-Recovered) phase plane is divided into 2 parts - one where the susceptibles need to isolate and another where they can get back to work. If the number of infected cases is high, the susceptible population should isolate to reduce the infection spread. For simplicity, the I-R plane is divided by a line, though better classification boundaries may exist. On one side of the line, the contact rate for the susceptible population is set to the minimum while on the other side, it is equal to the baseline (corresponding to opening the system).

The optimal slope of the dividing line is found via a *genetic algorithm* (Whitley., 1994) using *Matlab's* built-in optimization function *ga*, with the maximum generation number (set to 30) serving as the stopping criterion (MathWorks, 2020). The intercept of the line is fixed to a small negative number (10^{-3}) as the results are not affected by the intercept. The slope parameter is called θ, and the following cost function is minimized:

$$\mathcal{J}(\theta) = \widetilde{w}\frac{D(t_f)}{N_0} + \Psi(c),$$

where $D(t_f)/N_0$ gives the normalized casualties, $\Psi(c)$ is the fraction of working-days restored and $\widetilde{w}$ is a weight to bring the two terms on the same scale (see table S2). The output of the genetic algorithm is θ^*, the slope of the optimal dividing line. We assume that the divided plane on the side of the line with the origin, *i.e.,* zero infected and recovered cases, is the side where the susceptibles are free to work, while the divided plane on the other side corresponds to lockdown. The simulation is run for different isolation and shielding levels and the compiled results are shown in Figs. S9 and S10. The isolation efficiencies vary from 25% to 75% while the shielding ratio varies from 2 to 5.

We note that as the efficiency of isolation increases, the susceptible population can be let out for a longer duration towards the end of the epidemic. This is because the reduced contact rate of the infected population reduces the spread of infection. If the isolation efficiency is 65% or more, there is no need for the susceptible population to be in lockdown. Similar observations can be made for the shielding ratio. While the effects of it are not as dramatic, an increase in shielding ratio decreases the force of infection, resulting in a a lower time necessary for lockdown.

6. Sensitivity of optimal control and feedback control to mis-timed implementation of policies

We compute an optimal control for a system with a delayed input. We do not assume any modeling uncertainty, and attribute the perturbation only to the delayed application of the computed control. Suppose that an optimal control is computed for a scenario where its application is slated to commence 30 days after the outbreak of an epidemic, but the computed input control is uniformly delayed by 30 days. Then, by simulating the epidemic with a delayed policy, we can compare total deaths and working fraction for the delayed system with the results obtained without delay and with the same initial conditions.

The results, comprising total death and working fraction, are presented in table 1 for various isolation efficiencies. These findings indicate higher death rates as well as higher working fractions for the delayed system except for the third, and highest efficiency of isolation, where the respective performance measures are similar. For the case of 50% isolation, the death count is more than doubled due to delay. This significant difference in performance metrics demonstrates the shortcomings of implementing a policy based on optimal control if the system is not precise.

We also tested the sensitivities of the total death and working fraction to 30-day delays. The results, shown in table 2 in main text, indicate low sensitivity compared to the observed results for the optimal control solution. We compared these performance metrics obtained from applications of the optimal control vs. feedback control to the system without delay. The results, summarized in tables 1 and 2

respectively, indicate similar performance with respect to the performance metrics used. Thus, feedback control is nearly as good as optimal control if there is no delay; while having more robust features in the event that there is a delay in application.

7. Sensitivity of feedback control for mis-estimated isolation efficiency

Robustness of the feedback strategy (in terms of model mis-specification) can be studied by computing the total deaths and working fraction for the system with mis-specified isolation efficiency (c_{min}). In doing so, health and economic outcomes for the mis-specified system (i.e., overestimate or underestimate of isolation efficiency) are compared with the results obtained from the system with an accurately estimated isolation efficiency.

The isolation efficiency is varied from 35% to 75% with a 2% increment. For every isolation efficiency (for example with a true isolation efficiency of 50%), we consider three cases - unbiased, overestimated and underestimated isolation efficiency. For the unbiased case, i.e., when the isolation efficiency is accurate, we apply the feedback strategy (optimal line) associated with 50% isolation efficiency to the system to obtain the total deaths and working fraction. Second, for the case of overestimation, the isolation efficiency is assumed to be $\alpha\%$ higher, so we apply the feedback strategy associated with $(50 + \alpha)\%$ isolation efficiency to obtain the total deaths and working fraction in the overestimated scenario. Finally, we consider the underestimated case that is similar to the overestimated case, in which the feedback strategy associated with $(50 - \alpha)\%$ isolation efficiency is applied to the system.

The results are presented in Fig. S11. The feedback strategy is robust, especially when isolation efficiency is either low ($< 40\%$) or high ($> 65\%$). The results are expected as the feedback strategy is stationary in those ranges, i.e., when isolation efficiency is low, our policy suggests lockdown (for susceptible individuals) most of time; when isolation efficiency is high, our policy suggests staying open (for susceptible individuals), see Figs. S9 and S10. However, the feedback strategy is (slightly) sensitive when the isolation efficiency is in the range of $40\% \sim 65\%$. From 40% isolation efficiency to 65% isolation efficiency, lockdown policy (for susceptible individuals) switches to full open decision (for susceptible individuals), hence a small estimation error may cause to nonnegligible changes in policy, which may lead to the variability in working fraction and total deaths. As we can observe from Figs. S9 and S10, the feedback strategy varies rapidly from $40\% \sim 65\%$, i.e., from lockdown most of time to open entirely.
